# Systematic assessment of secondary bile acid metabolism in gut microbes reveals distinct metabolic capabilities in inflammatory bowel disease

**DOI:** 10.1186/s40168-019-0689-3

**Published:** 2019-05-15

**Authors:** Almut Heinken, Dmitry A. Ravcheev, Federico Baldini, Laurent Heirendt, Ronan M. T. Fleming, Ines Thiele

**Affiliations:** 1 School of Medicine, National University of Ireland, Galway, University Road, Galway, Ireland; 2 Luxembourg Centre for Systems Biomedicine, University of Luxembourg, Belvaux, Luxembourg; 3 Division of Analytical Biosciences, Leiden Academic Centre for Drug Research, Faculty of Science, University of Leiden, Leiden, The Netherlands; 4 Discipline of Microbiology, School of Natural Sciences, National University of Ireland, Galway, University Road, Galway, Ireland

**Keywords:** Gut microbiome, Bile acids, Host-microbe interactions, Metabolism, Genome-scale reconstruction, Constraint-based modeling, Personalized modeling, Systems biology

## Abstract

**Background:**

The human gut microbiome performs important functions in human health and disease. A classic example for host-gut microbial co-metabolism is host biosynthesis of primary bile acids and their subsequent deconjugation and transformation by the gut microbiome. To understand these system-level host-microbe interactions, a mechanistic, multi-scale computational systems biology approach that integrates the different types of omic data is needed. Here, we use a systematic workflow to computationally model bile acid metabolism in gut microbes and microbial communities.

**Results:**

Therefore, we first performed a comparative genomic analysis of bile acid deconjugation and biotransformation pathways in 693 human gut microbial genomes and expanded 232 curated genome-scale microbial metabolic reconstructions with the corresponding reactions (available at https://vmh.life). We then predicted the bile acid biotransformation potential of each microbe and in combination with other microbes. We found that each microbe could produce maximally six of the 13 secondary bile acids *in silico*, while microbial pairs could produce up to 12 bile acids, suggesting bile acid biotransformation being a microbial community task. To investigate the metabolic potential of a given microbiome, publicly available metagenomics data from healthy Western individuals, as well as inflammatory bowel disease patients and healthy controls, were mapped onto the genomes of the reconstructed strains. We constructed for each individual a large-scale personalized microbial community model that takes into account strain-level abundances. Using flux balance analysis, we found considerable variation in the potential to deconjugate and transform primary bile acids between the gut microbiomes of healthy individuals. Moreover, the microbiomes of pediatric inflammatory bowel disease patients were significantly depleted in their bile acid production potential compared with that of controls. The contributions of each strain to overall bile acid production potential across individuals were found to be distinct between inflammatory bowel disease patients and controls. Finally, bottlenecks limiting secondary bile acid production potential were identified in each microbiome model.

**Conclusions:**

This large-scale modeling approach provides a novel way of analyzing metagenomics data to accelerate our understanding of the metabolic interactions between the host and gut microbiomes in health and diseases states. Our models and tools are freely available to the scientific community.

**Electronic supplementary material:**

The online version of this article (10.1186/s40168-019-0689-3) contains supplementary material, which is available to authorized users.

## Introduction

The human gut microbiome performs essential functions for human health and is directly implicated in the pathogenesis of complex diseases, such as inflammatory bowel disease, obesity, and type II diabetes [1]. Since the etiology of these diseases is multifactorial, they can be seen as having a malfunctioning network rather than a single cause [1]. To understand the interplay between the factors underlying the disease network, such as genome, microbiome, and diet, computational systems biology approaches are necessary to integrate the different -omes, such as metagenome and metabolome, and to identify key interactions in an unbiased manner [1]. Such data-driven systems biology approaches could also identify drug-network interactions [1] and predict individual treatment responses in patients [1, 2].

One important function carried out by the human gut microbiome is the deconjugation of human primary bile acids and their subsequent biotransformation to secondary bile acids with implications for human health [3]. Briefly, the human liver synthesizes the primary bile acids cholate (CA) and chenodeoxycholate (CDCA), which are each conjugated with either taurine or glycine [4]. Conjugated bile acids are stored in the gall bladder and released into the small intestine after a meal [4]. In the intestine, they are subject to extensive metabolism by gut microbes, namely deconjugation of glycine or taurine, and biotransformation of the unconjugated primary bile acids to secondary bile acids [4]. Primary and secondary bile acids have endocrine functions and modulate host metabolism [3]; thus, their composition has important implications for human health. A link between microbial bile acid metabolism and inflammatory bowel disease (IBD), i.e., ulcerative colitis and Crohn’s Disease, has been repeatedly demonstrated [5]. In IBD patients, fecal conjugated bile acid levels are higher while secondary bile acid levels are lower and the deconjugation and transformation abilities of IBD-associated microbiomes are impaired [5]. Other diseases that have been associated with alterations of the intestinal bile acids pool include liver cirrhosis, liver cancer, irritable bowel syndrome, short bowel syndrome, and obesity [3, 6]; however, a mechanistic understanding of these bile acid-microbiome-disease associations is lacking. Thus, the role of bile acid composition and its relationship with the gut microbiome in these diseases needs to be elucidated and to be considered for therapeutic options [6].

A well-established computational approach for modeling human and microbial metabolism is Constraint-based Reconstruction and Analysis (COBRA) [7]. The COBRA approach relies on having genome-scale reconstruction of a target organism, which assembled based on the organism’s genome sequence and manually curated against the available genomic data and literature following established protocols [8]. A genome-scale reconstruction can readily be converted into a mathematical model, in which reactions and metabolites are represented as a stochiometric matrix, and interrogated using established methods such as flux balance analysis (FBA) [9]. Briefly, FBA relies on physicochemical (e.g., mass-charge balance) and environmental (e.g., nutrient uptake) constraints that limit the flow of metabolites through the network resulting in a solution space of feasible flux distributions [9]. Generally, FBA relies on the definition of an objective function, such as the biomass reaction, which sums all known precursors required to form a new cell. The objective function is then minimized or maximized, and the optimal solution, aka flux distribution, under the given condition-specific constraints is computed [9]. FBA operates under the steady-state assumption and as such does not require kinetic parameters to compute an optimal solution [9]. Through implementation of condition-specific constraints, e.g., a certain dietary regime, COBRA simulations have provided further insight into the metabolic capabilities of, e.g., human intestinal microbes [10–14], for which a comprehensive collection of reconstructions (AGORA) has been published [15, 16]. An advantage of the COBRA approach for microbial community modeling is that the underlying genome-scale metabolic networks enable mechanistic predictions of metabolic fluxes in each individual species while taking into account biological features, such as substrate availability or species-species boundaries [17, 18]. Previous studies have already demonstrated the use of constraint-based multi-species models for the prediction of host-microbe interactions [12, 19] and gut microbial community interactions [13, 20]. COBRA models can also be contextualized through omics data, e.g., metagenomic data [2, 14]. More importantly, by mapping metagenomic data of an individual, the metabolic microbial community model is personalized to this individual enabling the prediction of personalized metabolic profiles, which can be used to ultimately stratify disease and control groups [2, 14].

## Results

**Fig. 1:**
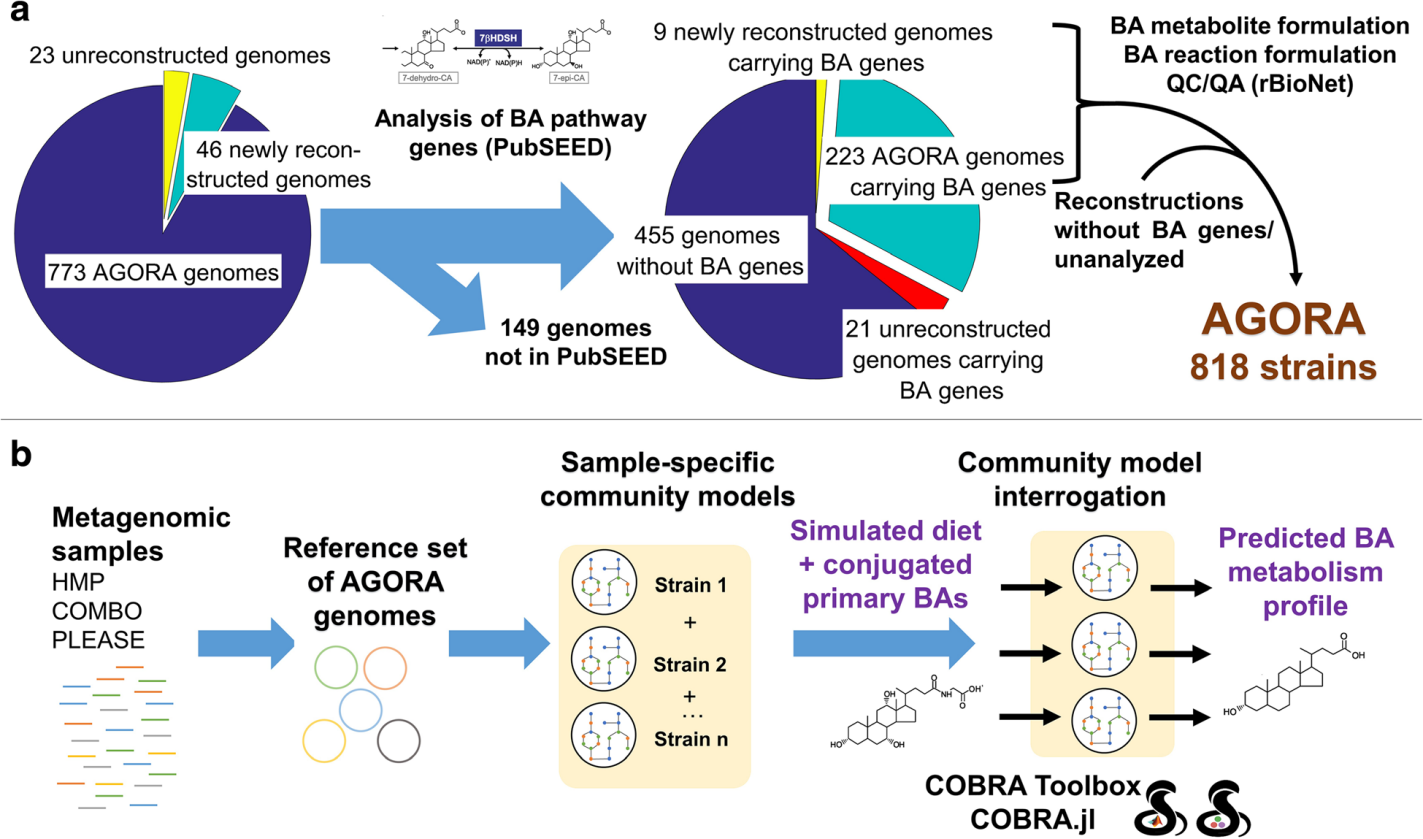
Schematic overview of the workflow in this study. **a** Comparative genomic and metabolic reconstruction approach used to expand the AGORA [15] resource with a bile acid (BA) deconjugation and biotransformation subsystem. The comparative genomic approach was performed in the PubSEED [26, 27] platform. Quality controland quality assurance (QC/QA) during reaction and metabolite formulation and addition to the AGORA reconstructions were ensured by using the reconstruction tool rBioNet [74]. **b** Computational pipeline used to predict the sample-specific bile acid deconjugation and biotransformation by human gut microbiomes. First, publicly available metagenomic data was retrieved from HMP [35], and the COMBO/PLEASE [36, 37] cohort. Next, the strain-level abundances were mapped onto the reference set of AGORA genomes. Microbial community models were constructed using the illustrated workflow, as implemented in the Microbiome Modeling Toolbox [33], and they account for the strain-level composition of each individual microbiome. Finally, each personalized community model was constrained with an “Average European” diet supplemented with conjugated primary bile acids and its individual-specific, primary bile acid deconjugation and biotransformation potential was computed using flux balance analysis [9, 76].

To investigate the microbiome-level bile-acid production potential of healthy individuals and IBD patients, we derived a systematic, reproducible workflow (Fig. 1). First, we expanded bile acid metabolism pathways captured in 232 gut microbial reconstructions using state-of-the-art comparative genomics methods. We then joined these reconstructions into pairwise microbial models and predicted their potential to cooperatively produce secondary bile acids. While each microbe could only produce up to six of the 13 secondary bile acids in silico, microbial pairs could produce up to 12 of the 13 bile acids, highlighting bile acid biotransformation as a microbial community task. Subsequently, we constructed functional and personalized gut microbiome models using metagenomics data from healthy and IBD individuals to predict an individual’s bile acid biosynthesis potential. We found inter-individual variation in the production capability of bile acids in healthy individuals as well as significant differences between healthy and IBD microbiomes. Moreover, we were able to compute the contribution of each strain to bile acid deconjugation and transformation while taking the metabolic network of the whole microbiome community and the applied constraints (e.g., dietary uptake) into account. Finally, we identified bottlenecks limiting the biotransformation potential into secondary bile acids. This mechanistic, microbiome-wide modeling approach can be readily applied to the personalized computation of other health-relevant human-microbial co-metabolites.

### Distribution of microbial bile acid deconjugation and biotransformation pathways across taxa

**Fig. 2:**
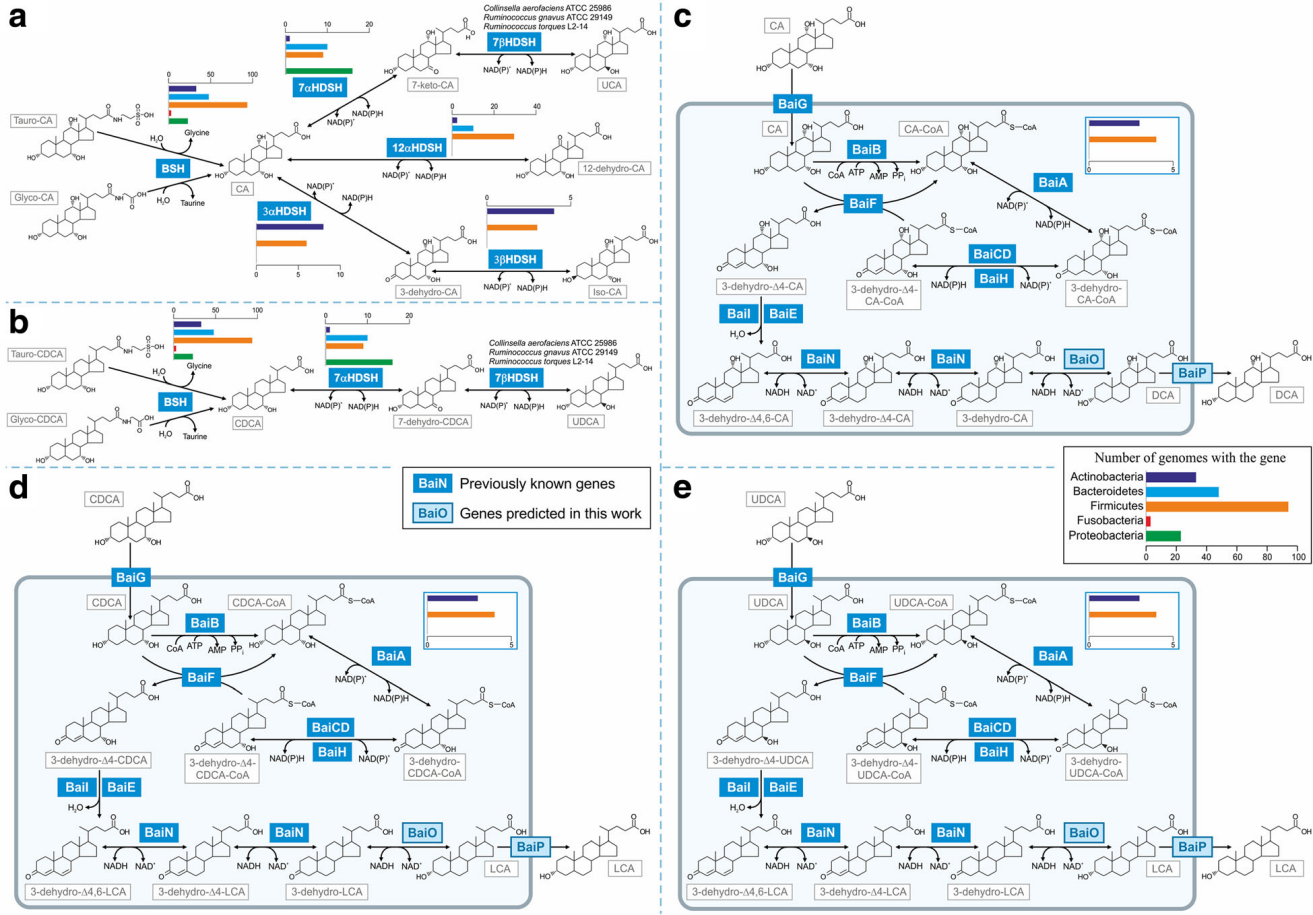
Illustration of bile acid pathways in human gut microbes reconstructed for AGORA. **a** Deconjugation of Tauro-CA/Glyco-CA and subsequent conversion to 12-dehydro-CA, UCA, and Iso-CA. **b** Deconjugation of Tauro-CDCA/Glyco-CDCA and subsequent conversion to UDCA. **c** Conversion of CA to DCA via the *bai* pathway. **d** Conversion of CDCA to LCA via the *bai* pathway. **e** Conversion of UDCA to LCA via the *bai* pathway. CoA Coenzyme A. For metabolite abbreviations, see Table 1.

To determine how widely genes encoding for bile acid pathways are spread in human gut microbes, we performed a systematic comparative genomic analysis of the bile acid deconjugation and transformation pathway (Fig. 2), starting with previously characterized enzymes for primary bile acid deconjugation [21] and transformation into secondary bile acids [22–25]. Of the currently 818 microbial AGORA reconstructions, which include 46 newly reconstructed gut microbes (see the “Materials and methods” for details), only 670 genomes were available at the PubSEED database [26, 27]. We additionally analyzed 23 further microbial genomes, yielding a total of 693 considered genomes (Fig. 1a). We found the bile salt hydrolase (*bsh*) gene, which encodes the deconjugation of conjugated primary bile acids, in 204 of the 693 (29%) genomes, including two archaeal genomes, *Methanobrevibacter smithii* ATCC 35061 and *Methanosphaera stadtmanae* DSM 3091 (Additional file 1: Table S1). The distribution of the *bsh* gene in Actinobacteria, Bacteroidetes, Firmicutes, as well as the two archaea (Fig. 2, Additional file 2: Figure S1) was in line with previously reported results [21]. Additionally, the *bsh* gene was found in 22 Proteobacteria genomes (Additional file 1: Table S1). Among all analyzed hydroxysteroid dehydrogenases (HSDHs), 7α-HSDH was the most widespread enzyme as it was found in 46 of the 693 (7%) genomes (Additional file 1: Table S1 and Additional file 2: Figure S2). Additionally, 3α-, 3β-, and 7β-HSDHs were found in 17, 12, and 3 genomes, respectively (Additional file 1: Table S1 and Additional file 2: Figure S2). Using results from a recent work [24], we found the 12α-HSDH in 39 genomes, which belonged mostly to Firmicutes representatives (Additional file 1: Table S1). We could not find the 12α-HSDH in the *Clostridium leptum* genome, although the enzymatic activity has been demonstrated [28].

The bile acid-inducible (*bai*) gene cluster for the multistep 7α/β-dehydroxylation pathway, which has been reported for *Clostridiaceae* and *Eggerthella* spp. [4, 23], was found in seven analyzed genomes belonging to *Clostridioides* sp., *Lachnoclostridium* sp., and *Eggerthella* sp. (Additional file 2: Figure S2). Remarkably, all these genomes also have genes for 12α-HSDH as well as either 7α-HSDH or genes for both 3α- and 3β-HSDHs (Additional file 1: Table S1). Thus, these microbes could play a crucial role in biotransformation of bile acids in the human intestine. The genes encoding for the last two steps of the 7α/β-dehydroxylation pathway, i.e., the NADH-dependent reduction and the export of secondary bile acids (Fig. 2c–e), have not been identified. Recently, the *baiN* gene was shown to encode a bi-functional enzyme NADH-dependent ∆6/∆4-hydroxysteroid reductase (Fig. 2) [29]. We analyzed the genomic context of this gene and found it in the *C. scindens* genome to be chromosomally co-localized with the gene for a probable NAD(FAD)-utilizing dehydrogenase (*CLOSCI_00522*). This chromosomal clustering was conserved in all Clostridiales genomes having the *bai* pathway, except for *Clostridium hiranonis* DSM 13275 (Additional file 2: Figure S3). It has been previously shown that genes encoding enzymes for the same metabolic pathway are often clustered on chromosome and such a clustering is conserved in genomes of related organisms [30]. Thus, the genes *baiN* and *CLOSCI_00522* can possibly belong to the same metabolic pathway, \namely *bai* pathway. The enzyme for the last step of the *bai* pathway, NADH-dependent 3α-hydroxysteroid reductase, is unknown. Because the product of *CLOSCI_00522* is a NADH-depended reductase, we propose that product is an enzyme catalyzing the final reaction of the *bai* pathway (Fig. 2) and rename the gene to *baiO*. The *bai* pathway has also been found in *Eggerthella lenta* [23]; consequently, we searched for orthologs of the *baiNO* genes in the *E. lenta* genome. Because *C. scindens* and *E. lenta* belong to different phyla, we defined orthologs of the analyzed genes as best/symmetrical bidirectional hits (see the “Materials and methods” section). An ortholog of the BaiN in *E. lenta* is likely to be encoded by the gene *Elen_1017* (protein identity = 32%, *e*-value for the protein alignment = 3e^−44^), whereas the BaiO ortholog was encoded by the gene *Elen_1018* (identity = 45%, *e*-value = e^−126^)*.* These genes were co-localized in *E. lenta*’s genome as well in genomes of *Eggerthella* sp. 1_3_56FAA and *Eggerthella* sp. HGA1 (Additional file 2: Figure S3). Additionally, these genes were co-localized with a gene encoding for a probable transporter (*Elen_1016*) in the genomes of *E. lenta* and *Eggerthella* sp. HGA1. Hence, the gene *Elen_1016* was assumed to encode a transporter for the products of the *bai* pathway and was named here *baiP*. An ortholog of this gene (*CLOSCI_01264*, identity = 59%, *e*-value = e^−180^) was found in *C. scindens* genome as well in other genomes of Clostridiaceae, having the *bai* pathway (Additional file 2: Figure S3), whereas in the *C. hiranonis* genome, this gene was co-localized with the *baiO* gene. Phylogenetic analysis of the BaiNOP proteins and their homologs revealed that BaiN and BaiO proteins of *Eggerthella* spp. and Clostridiales are phylogenetically distant from each other (Additional file 2: Figure S4 and S5), whereas BaiP proteins from these groups of genomes are phylogenetically close (Additional file 2: Figure S6).

In summary, our comparative genomics results expanded substantially our knowledge about bile acid deconjugation and transformation in gut microbes, while being consistent with previous studies [21–25]. Consequently, we propose that 253 of the 693 analyzed intestinal microbes (37%) can deconjugate and/or transform bile acids, including 232 reconstructed AGORA organisms (Additional file 1: Table S1, Fig. 1a).

### Expansion of the gut microbial genome-scale reconstructions by a species-specific bile acid subsystem.

The manual curation and refinement of genome-scale reconstructions is an iterative process [8]. Species-specific pathways are typically absent in draft reconstructions [31]. Since we did not explicitly account for bile acid pathways in the curation of AGORA [15] prior to the present paper, this subsystem was absent. The 232 metabolic reconstructions found to carry bile acid enzymes (Additional file 1: Table S1) were expanded with the corresponding metabolites and reactions, while ensuring functionality of the included pathways, following established procedures [8, 32] (see “Materials and methods” section). The complete reconstructed bile acid biotransformation subsystem contained 39 bile acid metabolites and 82 reactions (Fig. 2, Table 1, Additional file 1: Table S2a, b). For CA, CDCA, and the 13 secondary bile acids (Table 1), transport and exchange reactions enabling the uptake and secretion of these metabolites were added to the corresponding reconstructions. Taken together, we expanded the AGORA reconstructions with a bile acid module thus further improving their predictive potential and enabling their use for large-scale simulations of bile acid deconjugation and transformation.

**Table 1:** Overview of primary and secondary bile acids. *VMH* Virtual Metabolic Human database (https://vmh.life) [16]. AGORA, a compendium of 818 curated genome-scale gut microbial metabolic reconstructions used in this study.

Name	Abbreviation	VMH ID	Type	Producer
Taurocholate	Tauro-CA	tchola	Primary/conjugated	Human
Glycocholate	Glyco-CA	gchola		
Taurochenodeoxycholate	Tauro-CDCA	tdchola		
Glycochenodeoxycholate	Glyco-CDCA	dgchol		
Cholate	CA	cholate	Primary/unconjugated	Human; released by 185/818 AGORA strains
Chenodeoxycholate	CDCA	C02528		
12-dehydrocholate	12-dehydro-CA	12dhchol	Secondary	38/818 AGORA strains
7-ketodeoxycholate	7-keto-CA	7ocholate		41/818 AGORA strains
7-dehydrochenodeoxy-cholate	7-dehydro-CDCA	7dhcdchol		
3-dehydrocholate	3-dehydro-CA	3dhchol		16/818 AGORA strains
3-dehydrochenodeoxy-cholate	3-dehydro-CDCA	3dhcdchol		
Isocholate	Iso-CA	isochol		11/818 AGORA strains
Isochenodeoxycholate	Iso-CDCA	icdchol		
Lithocholate	LCA	HC02191		5/818 AGORA strains
Deoxycholate	DCA	dchac		
Allolithocholate	allo-LCA	alchac		
Allodeoxycholate	allo-DCA	adchac		
Ursocholate	UCA	uchol		3/818 AGORA strains
Ursodeoxycholate	UDCA	HC02194		

### Investigating the complementary capabilities of human gut microbes in silico

The majority of primary bile acids, released by the human gallbladder into the intestine, where the gut microbiome encounters them, are conjugated to glycine or taurine [3]. However, many strains capable of synthesizing secondary bile acids do not possess the bile salt hydrolase (Additional file 1: Table S1) and thus, rely on bile salt hydrolase-encoding strains to access the deconjugated primary bile acids.

To determine the capability of each strain alone to convert the deconjugated primary bile acids into secondary bile acids, the 232 corresponding AGORA reconstructions were converted into condition-specific models by applying an Average European diet supplemented with taurocholate (Tauro-CA), glycocholate (Glyco-CA), taurochenodeoxycholate (Tauro-CDCA), and glycochenodeoxycholate (Glyco-CDCA) (Additional file 1: Table S3) as modeling constraints. The maximally possible production flux for the 13 secondary bile acids was predicted for each strain using flux balance analysis [9] while setting the corresponding exchange reactions as the objective function (see the “Materials and methods” section). A total of nine strains could synthesize 7-ketodeoxycholate (7-keto-DCA) and 7-dehydrochenodeoxycholate (7-dehydro-CDCA) from the conjugated primary bile acids as they possessed both the bile salt hydrolase and the 7α-HSDH (Table 1). In contrast, no single strain was capable of synthesizing 12-dehydrocholate (12-dehydro-CA), ursocholate (UCA), and UDCA from the conjugated primary bile acids. Of the five strains carrying the *bai* gene cluster, only *Clostridium hiranonis* TO-931 could synthesize LCA and DCA from the conjugated primary bile acids (Table 1) as it also possessed the bile salt hydrolase enzyme in contrast to the other four strains. Taken together, only few strains could both deconjugate and biotransform primary acids in isolation.

**Fig. 3:**
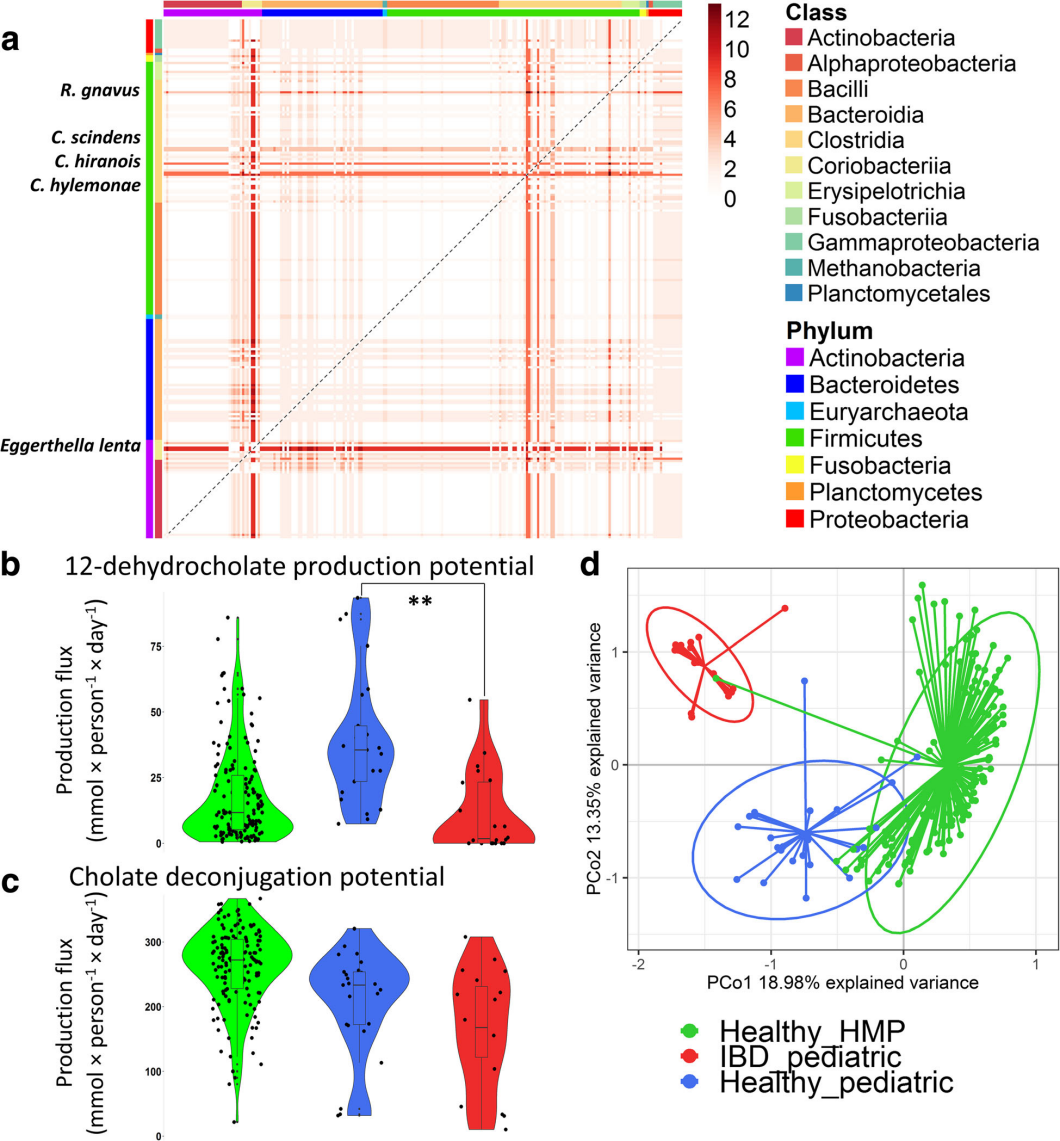
The predicted bile acid metabolic profiles of microbe-microbe pairs and individual gut microbiomes. **a** Complementary bile acid biosynthesis capabilities of the 232 gut microbial models with bile acid pathways joined in all possible combinations. The numbers of secondary bile acids (out of 13), which can be produced by each pair, are shown. **b**, **c** Total secretion potential in the healthy adults (Healthy_HMP), IBD patients (IBD_pediatric), and healthy pediatric controls (Healthy_pediatric) (flux values are given in mmol × person^-1^ × day^-1^): **b** deconjugated cholate, **c** 12-dehydro-CA. Significant difference (*p* value < 0.001) is indicated by stars. **d** Principal Coordinates Analysis of the strain-level contributions to two deconjugated primary and 13 secondary bile acids for the healthy adults, IBD patients, and healthy pediatric controls. Details on the strain to metabolite contributions are shown in Additional file 1: Table S8, and in Additional file 2: Figure S7.

To investigate whether pairwise combinations of certain strains could complement each other’s bile acid pathways, the 232 bile acid-producing AGORA models were joined in every possible combination resulting in 26,796 pairwise models using the Microbiome Modeling Toolbox [33], a COBRA Toolbox [34] extension. When comparing the bile acid production capabilities of the pairwise models with the respective single-strain models on the bile acid-supplemented “Average European” diet, we identified 7673 microbe pairs (29%) that could synthesize at least one secondary bile acid whereas the respective two individual strains were incapable to do so, resulting in 19,883 cooperative bile acid syntheses (Fig. 3a, Additional file 1: Table S5). For example, 3135/7673 pairs (40.9%) could synthesize 12-dehydro-CA from Glyco-CA or Tauro-CA (Additional file 1: Table S5). Further, 736 pairs (2.7%) and 100 pairs (0.4%) could synthesize DCA/LCA and UCA/UDCA, respectively, from the conjugated primary bile acids (Additional file 1: Table S5), demonstrating distinct bile acid synthesis capabilities of microbial pairs. There was no pairwise combination enabling synthesis of all secondary bile acids as the maximal number of secondary bile acids to be synthesized by any pair was 12 out of 13 (Fig. 3a). Taken together, while only few strains were capable of both bile acid deconjugation and biotransformation, many of the microbial pairs are predicted to synthesize secondary bile acids from the conjugated bile acids. This example demonstrates that constraint-based modeling is an efficient approach to elucidate the combined capabilities present in thousands of microbe pairs compared with the single microbes. The presented computational workflow could readily be applied to other microbial pathways of interest, in which enzymes are distributed across multiple taxa in an ecosystem.

### Large-scale modeling of the interpersonal variation in the bile acid deconjugation and transformation of gut microbiomes.

We next aimed to predict the bile acid deconjugation and biotransformation potential of individual-specific gut microbiomes. It is well known that bile acid metabolism is altered in individuals with IBD [3]. We were interested whether personalized modeling could provide novel insight into the differences in bile acid deconjugation and biotransformation potential between the microbiomes of IBD patients and controls as well as elucidate species contributing to the pathways. For comparison, we also evaluated the range in bile acid metabolic capabilities in healthy adults. We used metagenomic data from two sources: (1) 149 healthy American donors aged 18–40 years provided by the Human Microbiome Project Consortium [35] and (2) 20 children with newly diagnosed Crohn’s disease and microbial dysbiosis and 25 healthy controls (COMBO/PLEASE cohort [36, 37]). Using strain-level abundances, after mapping the reads onto the reference set of AGORA genomes [38], we generated personalized microbiome community models for each of the 194 sample by joining the corresponding metabolic reconstructions (Fig. 1b, the “Materials and methods” section ). Each microbiome model was constrained with the “Average European” diet supplemented with conjugated primary bile acids (Additional file 1: Table S3). A typical personalized microbiome model contained 127 AGORA models and 142,000 reactions (Table 2) making this work one of the largest constraint-based modeling efforts to date.

**Table 2:** Overview of the 194 personalized microbial community models generated within this study.

	Average number ± standard deviation	Minimum	Maximum
Microbes	127 ± 38	21	316
Reactions	141,727 ± 409,31	24,805	366,932
Metabolites	127,190 ± 371,22	21,967	328,108
Coupling constraints	200,618 ± 586,12	33,224	521,077

### The bile acid deconjugation and biotransformation potential is variable in healthy individuals and depleted in Crohn’s Disease patients

To predict the maximally possible bile acid deconjugation and biotransformation potential of the 194 microbiome models, we performed flux balance analysis while maximizing for the fecal secretion reaction flux (mmol × person^-1^ × day^-1^) of the primary bile acids, CA and CDCA, and 13 secondary bile acids (Fig. 3b, c and Additional file 1: Table S6). The quantitative production potential varied significantly between the models, with the quantitative production potential of LCA and DCA varying by a factor of 100 (Additional file 1: Table S6). A statistical analysis (Wilcoxon rank sum test, with *p* values adjusted for false discovery rate (FDR) by Benjamini-Hochberg method) was performed on the total community production potential of the 20 IBD patients (IBD_pediatric) and the 25 control microbiomes (Healthy_pediatric) (Fig. 3b, c, Additional file 1: Table S7). Compared with the microbiomes of healthy children, the IBD patient microbiomes were significantly depleted in 12-dehydrocholate production potential (*p* value < 0.001). Primary bile acid deconjugation potential was lower in IBD patients but only borderline significant after adjustment for FDR (adjusted *p* value = 0.0551); however, the abundance of the bile salt hydrolase reaction was significantly reduced in IBD microbiomes (*p* value 0.0235, Additional file 1: Table S7) and also differed based on phylum-and genus-level reaction abundances for many taxa (Additional file 1: Table S7). Microbiomes with low CA/CDCA liberation potential from the conjugated bile acids also had a low secondary bile acid potential (Additional file 1: Table S6) in agreement with the fact that the bile salt hydrolase is the gateway reaction in the pathway [39]. Taken together, we predicted the inter-person variability in the bile acid biosynthesis potential with microbiomes from IBD patients being significantly depleted in bile acid deconjugation and biotransformation potential, consistent with reports that IBD patients have higher levels of fecal conjugated and lower levels of secondary bile acids [5].

### Functional analysis of strain-level contributions in each microbiome.

What is the contribution of individual strains to the overall bile acid deconjugation and biotransformation potential? While previous studies have correlated certain taxa to measured metabolite levels [40], we determined here exactly which strains were producing the bile acids in the individual microbiome models using the aforementioned simulation results. Overall, 198 strains contributed to total production flux of at least one bile acid in at least one microbiome model (Additional file 1: Table S8). Of those, 15 strains contributed in > 90% of communities across both cohorts and thus play a significant role in bile acid metabolism. These strains included known commensals, such as *Ruminococcus gnavus* ATCC 29149, *Coprococcus comes* ATCC 27758, *Faecalibacterium prausnitzii* L2_6, *Clostridium* sp. M62_1, *Eubacterium ventriosum* ATCC 27560, *Bacteroides pectinophilus* ATCC 43243, and *Dorea formicigenerans* ATCC 27755. A variety of *Bacteroides* strains performed bile acid deconjugation and 7-keto-DCA/7-dehydro-CDCA biosynthesis, and their contribution was significantly depleted in the IBD microbiomes (*p* values for all < 0.01, Additional file 1: Table S7). Consistently, a positive correlation between *Bacteroides* spp. and secondary bile acid biosynthesis was found [36]. Using a Principal Coordinates Analysis on the strain-level contributions, we observed a clear separation between the IBD patients and controls (Fig. 3d), as well as between the HMP individuals and the pediatric individuals, due to the difference in strains (Fig. 3d, Additional file 2: Figure S7). The strain difference is most likely due to differences in age, location, and ethnicity of the two cohorts. On the phylum level, the contributions in both the healthy adult and healthy pediatric microbiomes were mostly driven by Actinobacteria, Bacteroidetes, and Firmicutes representatives, as expected, while Proteobacteria contributed significantly in the IBD microbiomes (*p* values for all < 0.05, Additional file 1: Table S7 and Additional file 2: Figure S7). A Wilcoxon rank sum test adjusted for FDR on strain-level contributions revealed that 303 strain-level contributions differed significantly between dysbiotic IBD and healthy pediatric microbiomes (*p* value < 0.05, Additional file 1: Table S7). Strain contributions mostly depleted in the IBD microbiomes included those of *Bacteroides vulgatus* ATCC 8482, *Ruminococcus* (*Blautia*) *torques* L2-14, *Faecalibacterium prausnitzii* strains, *Eubacterium rectale* strains*, Ruminococcus* sp. SR1-5, and *Clostridium* sp. M62-1 (*p* values for strains were < 0.001, Additional file 1: Table S7). The IBD microbiomes had significantly lower deconjugated CA and CDCA contributions by Actinobacteria and Bacteroidetes representatives (*p* values < 0.05, Additional file 1: Table S7 and Additional file 2: Figure S7). The contribution of 12-dehydro-CA production flux, which was significantly reduced in the IBD microbiomes (*p* value < 0.001, Additional file 1: Table S7) was attributed mostly to representatives of the *Lachnospiraceae* and *Ruminococcaceae* families, which are considered to be beneficial due to containing many butyrate producers [41]. These representatives included two *Faecalibacterium prausnitzii* strains (*p* value < 0.001), a species well known to be depleted in IBD [42]. In contrast, the overall 7-dehydro-CA production potential was comparable between the IBD and the healthy pediatric microbiomes (Additional file 1: Table S7). However, the strains contributing were different, with reduced contributions by commensal bacteria to the production of 7-keto-DCA/7-dehydro-CDCA, and increased contributions of the pathogenic *Escherichia coli* strains O157-H7 Sakai and UTI89 UPEC (Additional file 1: Table S7). These two strains contributed significantly higher to deconjugation and 7-keto-DCA/7-dehydro-CDCA production in the IBD microbiomes (*p* value < 0.001, Additional file 1: Table S7). Taken together, the dysbiotic IBD microbiomes, compared to the healthy control microbiomes, were depleted in contributions of a variety of commensal microbes to bile salt hydrolase and to bile acid biotransformation but enriched in contributions of pathogenic *Escherichia sp*. (Additional file 1: Table S7). Thus, the IBD microbiomes had distinct bile acid deconjugation and transformation potential, consistent with reports that bile acid composition in IBD patients is abnormal [5]. In total, 488 analyzed features, which encompasses total production, strain contributions, and reaction abundances, were significantly different (*p*-value <0.05), of which 375 were highly significant (*p*-value <0.001) (Additional file 1: Table S7). 

### Shadow price analysis identifies individual-specific bottlenecks in bile acid biotransformation potential.

**Fig. 4:**
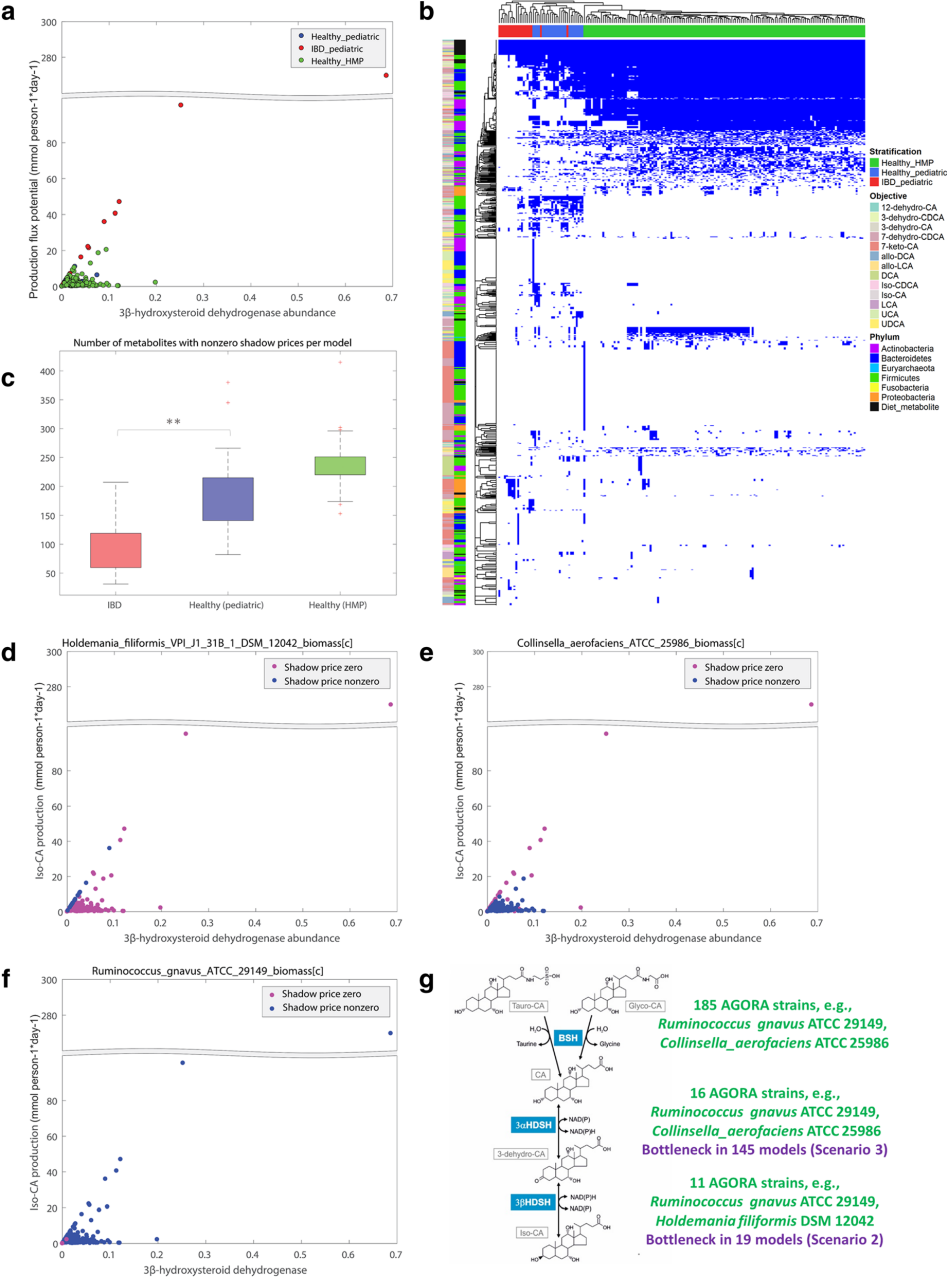
Metabolic bottlenecks and shadow price profiles computed when optimizing for Iso-CA production in 194 microbiome community models. **a** Abundance of the 3β-HSDH reaction yielding Iso-CA (VMH ID: ICA3bHSDHe) plotted against Iso-CA production potential (flux values are given in mmol gDW^−1^ h^−1^) for the 194 microbiomes. **b** Heat map of the shadow prices retrieved from the 194 microbiomes when the production of two deconjugated primary and 13 secondary bile acids was optimized. Blue and white data points show nonzero and zero shadow prices, respectively. The columns show the 194 microbiomes annotated by group. The rows show all metabolites that had a nonzero shadow price in at least one community model. The metabolites are annotated by taxonomy. Entries annotated with “Diet_metabolite” represent bile acid metabolites present in the dietary, luminal, or fecal compartment of the microbiome model. The “bile acid optimized” color bar shows the bile acid for which production was optimized. **c** Number of metabolites with nonzero shadow prices in the microbiome models of healthy adults, IBD patients, and healthy pediatric controls. Significant difference (*p* value < 0.001) is indicated by stars. **d** Shadow prices for the biomass metabolite of *Holdemania filiformis* DSM 12042, a strain carrying 3α-HSDH but not 3β-HSDH, plotted against Iso-CA production and abundance of the 3β-HSDH reaction. Blue dots indicate models belonging to Scenario 2 (see main text). **e** Shadow prices for the biomass metabolite of *Collinsella aerofaciens* ATCC 25986, a strain carrying 3β-HSDH but not 3α-HSDH, plotted against Iso-CA production and abundance of the 3β-HSDH reaction. Blue dots indicate models belonging to Scenario 3 (see main text). **f** Shadow prices for the biomass metabolite of *Ruminococcus gnavus* ATCC 29149, a strain carrying both 3α-HSDH and 3β-HSDH, plotted against Iso-CA production and abundance of the 3β-HSDH reaction. **g** Pathway for Iso-CA biosynthesis from Glyco-or Tauro-CA, and depiction of the steps representing bottlenecks in Scenarios 2 and 3 (see main text). For metabolite abbreviations see Table 1. For simplicity, sections of the *y* axis without any data are omitted in **a** and **d**–**f** (indicated by the two gray lines).

To test whether the bile acid production potential could be directly predicted from the abundance of the metagenomics data mapped onto the AGORA reconstruction (i.e., from the encoding gene abundance), we calculated the Spearman correlation between the individual production potential for the two deconjugated primary and 13 secondary bile acids (Table 1) and the total community abundance for all reactions in the bile acid pathway in the 194 community models. Consistently, for 13 of the 15 bile acids, the correlation between production potential and the abundance of reaction directly synthesizing the respective bile acid was 0.96 or higher (Additional file 1: Table S9). The secondary bile acid Iso-CA is synthesized from 3-dehydro-CA by 3β-HSDH. Surprisingly, the Spearman correlation between production potential for Iso-CA/Iso-CDCA and the abundance of the 3β-HSDH reaction (VMH ID: ICA3bHSDHe/ ICDCA3bHSDHe) calculated for all 194 microbiome models was only 0.18 indicating that the reaction abundance of the producing reaction did not correlate with production (Additional file 1: Table S9). In fact, only a minority of the 194 microbiome models with a high abundance of the 3β-HSDH reaction (VMH ID: ICA3bHSDHe), all of which were IBD microbiomes, also had a high Iso-CA production flux (Fig. 4a). Thus, factors other than 3β-HSDH abundance limited the production flux. To identify these factors, the metabolic fluxes needed to be analyzed in the context of the pathway and the microbial community. Constraint-based modeling is ideal for such analyses of metabolic dependencies since it is mechanistic on the molecule level and takes species-species metabolic exchanges and boundaries into account [18].

To identify the factors limiting the production potential for secondary bile acids, the shadow prices associated with the flux solutions of each microbiome model were analyzed. Shadow prices are a standard feature of constraint-based modeling that are routinely calculated with each feasible flux balance analysis solution. Briefly, the shadow price is a measurement for the value of a metabolite towards the optimized objective function, which indicates whether the flux through the objective function would increase or decrease when the availability of this metabolite would increase by one unit [7]. A positive or negative shadow price indicates that increased availability of the metabolite would either increase or decrease the flux through the objective function (note that this definition varies by solver), respectively. In contrast, the availability of a metabolite with a shadow price of zero has no influence on the flux through the objective function. To identify limiting factors for secondary bile acid production, we investigated the shadow prices in the flux balance analysis solutions (see the “Materials and methods” section) when optimizing the production of the secondary bile acids in the 194 microbiome models (Fig. 4b, Additional file 1: Table S10). Nonzero shadow prices with an absolute value higher than 10^−6^ indicating importance for bile acid production flux were found for biomass metabolites of 129 strains carrying bile acid enzymes, for strain-specific metabolites in the bile acid pathway, and for dietary exchange metabolites of the bile acids. Overall, 1138 microbial and dietary metabolites were found to be relevant for bile-acid synthesis in the entire set of microbiome models (Additional file 1: Table S10). When comparing the shadow prices in the three groups, the number of metabolites with nonzero shadow prices was significantly lower in the IBD microbiomes than either in the healthy pediatric or healthy adult microbiomes (Fig. 4c). Hence, the pediatric IBD patients were depleted in strains with bile acid biosynthesis capabilities. This result highlights that an increase in secondary bile acid biosynthesis in these individual communities could only be achieved by introducing additional microbial strains.

Next, we aimed to identify the factors limiting Iso-CA biosynthesis potential. Of the reconstructed strains, 16 and 11 strains, respectively, carried the 3α- and 3β-HSDH enzyme (Additional file 1: Table S1). Five strains (*Eggerthella* sp. 1_3_56FAA, *Eggerthella lenta* DSM 2243, *Gordonibacter pamelaeae* 7-10-1-bT, *Mycobacterium avium* subsp. *avium* ATCC 25291, and *Ruminococcus gnavus* ATCC 29149) possessed both enzymes. Of the strains possessing either or both enzymes, 18 were present in at least one of the 194 microbiome models. The shadow prices corresponding to Iso-CA production were inspected (Additional file 1: Table S10). Note that shadow prices for Iso-CDCA were analogous.

Four scenarios could be distinguished based on the shadow prices. Seven microbiomes belonging to the first scenario were unable to synthesize Iso-CA (Additional file 1: Table S6). Consequently, in these microbiomes, shadow prices were only nonzero for dietary Iso-CA (Additional file 1: Table S10) indicating that Iso-CA levels could only be increased by directly providing it. In the second scenario, which was found in 19 microbiomes, shadow prices for the six strains carrying 3β-HSDH but not 3α-HSDH were nonzero for at least one of the six strains’ biomass metabolites (Additional file 1: Table S10). In the same 19 microbiomes, shadow prices for all eight strains carrying 3α-HSDH but not 3β-HSDH were zero. This result showed that 3α-HSDH abundance was not a bottleneck and Iso-CA production could be increased by increasing the abundance of strains carrying 3β-HSDH. In these microbiomes the 3β-HSDH abundance directly correlated with Iso-CA production flux, as illustrated with the example of *Holdemania filiformis* DSM 12042 in Fig. 4d. In the third scenario, which contained the majority of microbiomes (145 cases), the shadow prices for all six strains carrying 3β-HSDH but no 3α-HSDH were zero. Instead, the shadow prices for at least one of the eight strains carrying 3α-HSDH but not 3β-HSDH were nonzero. Consequently, in these microbiomes, the availability of the precursor 3-dehydro-CA was flux-limiting and Iso-CA production could not be increased by increasing the abundance of strains carrying only 3β-HSDH. These 145 microbiomes had the lower than expected Iso-CA production potential (Fig. 4e). As expected, in all 145 microbiomes, the shadow price for dietary 3-dehydro-CA, the precursor of Iso-CA, was also nonzero (Additional file 1: Table S10). Finally, in the fourth scenario, which consisted of 22 microbiomes, shadow prices were nonzero only for the biomass metabolite of *Ruminococcus gnavus* ATCC 29149 and in some cases *Eggerthella lenta* DSM 2243 (Fig. 4f). These two strains possess both 3α-HSDH and 3β-HSDH and are present in most microbiomes in this study. Thus, they played a central role for all microbiomes’ capabilities to synthesize Iso-CA.

In summary, by analyzing the shadow prices associated with each flux balance analysis solution when optimizing for secondary bile acid production, strain-specific contributions to their biosynthesis were determined for each personalized community model. Four scenarios with different bottlenecks for the biosynthesis of Iso-CA were identified. This analysis highlights once more that the metabolic potential of an individual microbiome, and strategies to manipulate this metabolic potential, cannot be inferred solely from the abundance of single genes and depends on the community-wide metabolic network as well as metabolic constraints (e.g., substrate availability). We demonstrated that constraint-based modeling allows for the generation of mechanistic, testable hypotheses.

## Discussion

In this work, we used a systematic computational modeling workflow to investigate the bile acid production capabilities of gut microbes and gut microbial communities. After annotating and reconstructing the bile acid deconjugation and transformation pathways (Fig. 1a, Fig. 2) in 693 human gut microbe genomes, we first built pairwise microbial models providing novel insight into strain-specific bile-acid production capabilities. We then assembled gut microbiome models for each metagenomics sample of either healthy individuals or pediatric IDB patients. The three key results of our analysis are as follows: (1) microbes can complement each other’s bile acid pathway to achieve the broader bile acid production repertoire observed in fecal samples, (2) bile acid production profiles of 194 microbiome models were individual-specific and distinguished healthy controls from pediatric IBD patients, and (3) the bile acid production profiles could not be predicted by reaction (gene) abundance alone, as illustrated for Iso-CA illustrating the added value of computational modeling of metabolite production capabilities of microbial communities.

While it can be intuitively understood that bile acid biosynthesis is a cooperative task in the gut microbiome from the known fact that no strain possesses the complete pathway [23], these microbe-microbe metabolic dependencies could be exactly predicted through constraint-based modeling yielding more than 7000 pairs of microbes (Fig. 3a, Additional file 1: Table S5). The capabilities of most strains to generate secondary bile acids were shown to be very limited. For example, no strain alone but 100 pairs could convert tauro-or glyco-CDCA into UDCA (Additional file 1: Table S5). This analysis demonstrated that strain-specific microbe-microbe interactions need to be considered when studying the metabolic crosstalk between the gut microbiome and the mammalian host. Similar microbial corporations through cross-feeding of metabolic products have been suggested, e.g., for intestinal microbial metabolism of b-vitamins [43], of host-derived mucins [44], of dietary glycans [45], of flavonoids [46], for short-chain fatty acid production [41], and for microbial respiratory capabilities [47].

The personalized bile acid metabolism profile of 194 microbiomes, which included the total production potential and the strain-level contributions to overall production was individual-specific and distinct from healthy controls in pediatric IBD patients (Fig. 3b–d, Additional file 2: Figure S7). Our finding that the bile acid profiles of IBD patients differ from healthy controls agrees with experimental reports. For instance, a recent study has investigated the microbiomes and fecal metabolomes of pediatric IBD patients and their relatives and could distinguish two metabotypes both in patients and relatives [48]. The IBD-associated metabotype has been characterized by an altered bile acid profile, with increased levels of cholate and sulfated and taurine-conjugated primary bile acids. The altered bile acid profile suggests a reduced bile acid deconjugation and conversion potential of the gut microbiota [48], which we could demonstrate being the case with our in silico results (Fig. 3b–c).

In most analyzed microbiome models, the production potential for Iso-CA was found to be lower than expected from the abundance of the 3β-HSDH. Analyzing the shadow prices [7] revealed that the presence of strains capable of synthesizing the precursor 3-dehydro-CA was a bottleneck in many microbiomes. In fact, we identified four scenarios, for which different strategies could be used to increase overall Iso-CA production capabilities in a given microbiome. In these four scenarios, Iso-CA production flux could be increased (1) only by directly providing it, (2) by increasing the abundance of strains carrying 3β-HSDH, (3) either by providing 3-dehydro-CA or by increasing the abundance of strains carrying 3α-HSDH, and (4) by increasing the abundance of *Ruminococcus gnavus* ATCC 29149 and in some cases *Eggerthella lenta* DSM 2243. To complete the systems biology cycle, these predictions require experimental validation, e.g., by measuring the amount of Iso-CA levels in in vitro cultures from fecal samples. A shadow price analysis has the advantage of being an unbiased indicator for metabolites in a pathway that are of key importance for the end product of the pathway. It could be readily applied to other health-relevant metabolites produced by the gut microbiome (e.g., short-chain fatty acids) and key synthesis-limiting steps in the relevant pathways.

Compared with commonly used computational and multivariate statistical approaches, the constraint-based modeling approach applied in this study has several key advantages. First of all, unlike quantifications of total gene abundance (e.g., [49]) and correlation-based approaches (e.g., [50]), our approach is mechanistic and obeys physicochemical and environmental constraints (e.g., mass-charge conservation, laws of thermodynamics, substrate uptake). This property enabled us to predict the metabolic capabilities of a given microbial community, as defined by metagenomics data. Importantly, the predicted capabilities are physiologically, physicochemically, and thermodynamically feasible under the given medium conditions (i.e., diet). Second, the metabolic reconstructions used in our approach are strain-resolved, and the capabilities included in each metabolic network are based on the microbes’ genome, detailed comparative genomic analyses as well as an extensive review of the literature for biochemical and physiological data [15]. As a consequence, the metabolic contribution of each strain in each individual microbiome can be exactly predicted with high confidence. Another advantage of our approach is the incorporation of species-species boundaries and transport capabilities. As stated above, species-species cross-feeding plays a key role for the metabolic potential of a microbial community and thus needs to be considered. Finally, it is challenging to link typical metagenomics-based approaches to a particular host function. Microbial species or functions can be correlated with certain host metabolites through top-down multivariate statistical analyses [50]. However, mechanisms explaining these correlations are often lacking. As more omics data become available for microbiome samples, the generated microbiome models can be further constrained and personalized through the integration of meta-transcriptomic [51], meta-metabolomic [52], meta-proteomic data [53], or nutritional information via the Virtual Metabolic Human database [16]. The microbiome models can also be integrated with the global human reconstruction, Recon3D, which includes a secondary bile detoxification subsystem [54], or with the whole-body organ-resolved reconstruction of human metabolism [19] thanks to the use of a consistent namespace [15]. The integrated analysis can predict organ-specific metabolic changes due to differences in microbial community composition and yield novel hypotheses about host-microbiome co-metabolism [19].

One limitation of the method is the steady-state assumption of flux balance analysis and the resulting computation of fluxes rather than concentrations. Moreover, the AGORA reconstructions and our modeling framework do not include regulatory constraints and kinetic parameters. As a result, the modeling framework does not account for substrate specificity and transporter capacity, although the latter could be incorporated as reaction constraints dependent on data availability. This limitation could be overcome using hybrid modeling techniques that integrate the dynamics and the regulation of biochemical processes through with differential equations [55–58]. Furthermore, our method does not allow predicting microbial composition or organismal abundances in the microbiome, again due to the steady-state assumption. The method relies on parameterizing the personalized models with the relative microbial abundances calculated from the metagenomic data. For predicting microbial abundances, dynamic community flux balance analysis methods [58, 59] are more appropriate. Consequently, we focus the application of our framework on exploring the metabolic profile of a given gut microbiome with known microbial composition. Finally, it is well known that the gut microbiome fluctuates over time [60], however, each simulation performed with the personalized models only represents the fecal microbiome at a single time point. This is expected as the fecal metagenomic sample that serves as the input data also only captures the gut microbiome at a single time point. Fecal metagenomic samples from the same individuals at multiple time points are, for example, available in [61]. Such data could be used to model a time series of metabolic states and elucidate how the gut microbial metabolic profiles fluctuate over time.

Flux profiles predicted by the framework can be readily compared with qualitative increases or decreases in metabolites in disease conditions to validate simulation results, which would require metagenomic or 16S rRNA data as well as fecal metabolomics from the same subjects. Metagenomic and fecal metabolomic measurements of bile acids have been performed in [62] and such data could be linked through modeling in future efforts. Such comparisons have valuable applications for mechanistically linking metagenomic and metabolomic measurements from the same sample. Moreover, qualitative and quantitative metabolomic data could be used as input data to contextualize the models further. A COBRA Toolbox module for the implementation of metabolomic data with constraint-based models has been developed [52].

While the scope of the present work is the prediction of bile acid metabolism, in future efforts, other health-relevant microbial metabolic subsystems may be considered. For instance, Lewis et al. found that several pathways, e.g., glycerophospholipid metabolism, amino benzoate degradation, sulfur relay system, and glutathione metabolism, separated healthy and dysbiotic microbiomes [36]. In a follow-up work, fecal amino acid levels have been found to be altered in IBD patients and to positively correlate with Proteobacteria [40]. Applying the computational workflow presented in this study to predict the gut microbial metabolome beyond bile acid metabolism would allow us to mechanistically link altered metabolites with strain-specific capabilities. Ultimately, such analysis could lead to novel insights into the mechanisms behind altered metabolomes in disease states and allow pinpointing disease-relevant species and/or enzymes that may serve as novel drug targets.

## Conclusions

We demonstrated that an in silico metabolic modeling workflow could elucidate the metabolic potential of an individual’s microbiome, which cannot be done based on gene count and reaction abundance alone. We illustrated this workflow using metagenomics data of healthy individuals and IBD patients while focusing on bile acid metabolism. Importantly, we were able to demonstrate that this mechanistic, strain- and metabolite-resolved, unbiased, and inexpensive approach allows for the systematic interrogation of the metabolic potential of an individual’s microbiome and can yield testable novel hypotheses. Integrative systems biology approaches are urgently needed to gain novel insight into complex, multifactorial diseases, such as IBD [1]. In future efforts, personalized modeling could also be applied to predicting individual-specific dietary or therapeutic interventions [63]. The AGORA resource, the COBRA Toolbox, and the Microbiome Modeling Toolbox are freely available to the scientific community. We have also created extensive tutorials (available at the COBRA Toolbox GitHub) aiding users interested in applying our framework. We expect that the metabolic modeling approach presented will have valuable applications in unraveling the role of human-gut microbiome metabolic interactions in human health and disease.

## Materials and methods

### Comparative genomic approach

All 773 strains of the AGORA resource [15], 46 strains reconstructed in this study, and 23 currently not reconstructed strains were analyzed for the presence of their genomes at the PubSEED resource [26, 27], resulting in 690 bacterial and three archaeal genomes to be considered in this study (Fig. 1a). Note that only 670 of the reconstructed microbes had their genomes available in PubSEED and were consequently used for the comparative genomic approach. All 693 human gut microbe genomes were analyzed for the presence of orthologs of bile acid deconjugation and biotransformation genes (Additional file 1: Table S1). Orthologs are defined as genes that satisfy the following conditions: (1) Orthologs should be closely homologous proteins (*e*-value cutoff = e−50). (2) Orthologs should be found in the same genomic context, i.e., the structure of gene locus should be conserved in related genomes. (3) Orthologs should form a monophyletic branch of a phylogenetic tree.

For the search of homologs and analysis of genomic context, the PubSEED platform was used along with phylogenetic trees for protein domains in MicrobesOnline [64]. Multiple protein alignments were performed using the MUSCLE v. 3.8.31 tool [65, 66]. Phylogenetic trees were constructed using the maximum-likelihood method with the default parameters implemented in PhyML-3.0 [67]. The obtained trees were visualized and midpoint-rooted using the interactive viewer Dendroscope, version 3.2.10, build 19 [68].

The following previously analyzed genes were used as a starting point: (1) genes for bile salt BSH from multiple genomes [21], (2) 7α–HSDH) from *Bacteroides fragilis* [22], (3) 3α- and 3β-HSDHs genes from *Eggerthella lenta* DSM 2243 and *Ruminococcus gnavus* ATCC 29149 [23], (4) 7α-HSDH and *baiABCDEFGHI* genes for a multistep 7α/β-dehydroxylation pathway, (5) *bai* genes from *Eggerthella lenta* DSM [23], (6) 7β-HSDH gene from *Clostridium absonum* [25], and (7) 12α-HSDH from *Clostridium hylemonae* DSM 15053, *Clostridium scindens* ATCC 35704, and *Clostridium hiranonis* DSM 13275 [24]. Note that *Clostridium leptum* has been experimentally shown to have 12α-HSDH activity [28]; however, we were unable to identify the 12α-HSDH gene in its genome. BSH proteins are closely related to the penicillin V amidase (PVA) proteins [21]. To avoid mis-annotations, a phylogenetic tree for BSH proteins and their homologs in the analyzed genomes was constructed (Additional file 2: Figure S1), and orthologs of the known BSH genes were identified. All HSDH proteins listed above demonstrated similarity to each other and with BaiA proteins. Thus, orthologs for HSDH/BaiA proteins were resolved through the construction of a phylogenetic tree (Additional file 2: Figure S2). Finally, two new genes in the 7α/β-dehydroxylation pathway (BaiO and BaiP, Fig. 2) were predicted in this work. All of the annotated genes are represented as a subsystem at the PubSEED website [69] and can be found in Additional file 1: Table S1.

### Formulation and addition of reactions

Reaction mechanisms were retrieved from the KEGG database [70] as well as published literature (e.g., [71]). For all genomes having genes for BSH, HSDHs, or the complete 7α/β-dehydroxylation pathway (Fig. 2), metabolic mass- and charge-balanced reactions were formulated. Exchange reactions were added for all extracellular metabolites. Most reactions were associated with genes and proteins annotated in the analyzed genomes. Reactions not-associated with genes and proteins were only added if the gene was unknown but the reaction was required to eliminate dead-ends in a metabolic pathway. Thus, the following gap-filling reactions were added without associations with genes or proteins: (1) A transport reaction for LCA, the final product of 7α/β-dehydroxylation pathway which was added as the transporter is unknown. (2) Pathways that yield allolithocholate (allo-LCA) and allodeoxycholate (allo-DCA) were included for strains possessing the *bai* gene cluster as these compounds are known to be side-products of the 7α/β-dehydroxylation pathway [72] and found in human adults under certain circumstances [3].

Pathways for cholesterol reduction to coprostanol were also reconstructed. These enzymatic activities, both cytoplasmic and extracellular, have been shown in *Lactobacillus acidophilus*, *Lactobacillus bulgaricus*, and *Lactobacillus casei* [73]. The precise mechanisms of these reactions as well as the enzyme-encoding genes are unknown, but the biotransformation has been shown to be associated with the oxidation of NADH to NAD^+^ [73]. Consequently, reactions for extracellular and cytoplasmic NADH-dependent reduction of cholesterol to coprostanol were added to six *Lactobacillus* sp. models, together with exchange reactions for cholesterol and coprostanol as well as a predicted transport reaction for cholesterol uptake.

All metabolites and reactions were formulated following an established reconstruction protocol [8]. Metabolites and reaction abbreviations in the bile acid subsystem were created in accordance with the Virtual Metabolic Human (VMH) [16] nomenclature to ensure compatibility with the human metabolic reconstruction. The MATLAB-based reconstruction tool rBioNet [74], which ensures quality control and quality assurance, such as mass- and charge-balance, was used to add the metabolites and reactions to the appropriate reconstructions. All reactions and metabolites in the reconstructed bile acid subsystem are described in Additional file 1: Table S2a, b.

### Expansion of AGORA

A total of 46 gut microbial strains were newly reconstructed. The reconstructions were generated by semi-automatically expanding and curating KBase [75] draft reconstructions following the established AGORA pipeline used in [15] (Additional file 1: Table S11). Of the 773 AGORA strains and 46 newly reconstructed strains, 232 strains total carried at least one gene in the bile acid pathway (Additional file 1: Table S1) and six produced coprostanol. The corresponding reconstructions were expanded by the appropriate metabolites and reactions using rBioNet [74] and subjected to extensive quality-assurance and control measures [8, 32] (Fig. 1a). The expanded resource, accounting for 818 strains, is available on the Virtual Metabolic Human website [16].

### Construction of pairwise models

The 232 AGORA reconstructions carrying bile acid reactions were joined pairwise in every possible combination as described previously [15] using the Microbiome Modeling Toolbox [33]. In total, 26,796 pairwise models were created.

### Construction of sample-specific gut microbiota models

Metagenomic datasets from 194 samples in total were obtained from three sources: (1) Strain-specific relative abundance data from 149 individual microbiotas of healthy American individuals was obtained from the Human Microbiome Project website [35]. (2) Paired-end Illumina raw reads of 33 dysbiotic Crohn’s Disease patients in the PLEASE cohort [36] and of 26 healthy controls in the COMBO cohort [37] were retrieved from NCBI SRA under SRA: SRP057027. For the latter dataset, the reads had been pre-processed and then mapped onto the reference set of 773 AGORA genomes, as described in [38]. To reduce the number of false positives, a cutoff of 10% genome coverage was applied to the resulting coverages (representing a threshold of at least 10% genome coverage for each microbe in each human individual). The resulting coverages were normalized for each individual in order to obtain the relative abundances. To avoid too small model sizes, microbiome models, for which less than 20 strains could be mapped to the reference set of AGORA genomes, were excluded from the analysis. This was the case for 13 samples from the PLEASE cohort and one sample from the COMBO cohort.

Personalized microbiome models were then created using the mgPipe module in the Microbiome Modeling Toolbox [33] (Fig. 1b). Briefly, for all strains identified in at least one metagenomics sample, the corresponding AGORA reconstructions, if available, were joined into one global constraint-based microbiome community reconstruction as described elsewhere [17, 33]. For each of the 194 metagenomic samples, the list of all the mapped strains and their strain-level abundances served as input data for deriving a personalized microbiota model from the global community reconstruction, which consisted of the joined AGORA reconstructions corresponding to each strain present in the sample. The flux through each AGORA strain’s model was coupled to its respective biomass objective function, as described elsewhere [11]. Then, we parameterized the community biomass reaction by applying the strain-level abundances as stoichiometric values for each microbe biomass reaction in the community biomass reaction (Fig. 1b). These constraints enforced that all strains grew at the experimentally measured ratios. Subsequently, an Average European diet supplemented with conjugated primary bile acids (see below) was applied as constraints on the dietary exchanges. To simulate a realistic turnover of microbial biomass, the allowed flux through the community biomass reaction was set to be between 0.4 and 1 mmol × person^-1^ × day^-1^, corresponding to a fecal emptying of once every three days to once a day. The features of the personalized community models are given in Table 2.

### Definition of the average European diet

A diet representing the nutrient intake of an average European individual was obtained from the nutrition resource in the Virtual Metabolic Human database [16] along with the corresponding flux values. The diet was supplemented with metabolites previously determined necessary for the biomass production of at least one AGORA reconstruction [15]. Each microbiota community model was constrained with the Average European diet using a dedicated function in the Microbiome Modeling Toolbox [33] (adaptVMHDietToAGORA.m). Additionally, to enable modeling of bile acid transformation, the uptake of the conjugated primary bile acids Glyco-CA (VMH ID: gchola), Tauro-CA (VMH ID: tchola), Glyco-CDCA (VMH ID: dgchol), and Tauro-CDCA (VMH ID: tdchola) was allowed to be taken up unlimitedly by setting the lower bounds on the corresponding exchange reactions to − 1000 mmol × person^-1^ × day^-1^. The lower bounds on all other dietary exchange reactions were set to zero preventing the uptake of these metabolites. The constraints implemented to simulate the diet are given in Additional file 1: Table S3.

### Interrogation of models for bile acid synthesis capabilities

The bile acid production potential in 232 AGORA reconstructions and 26,796 pairwise models was determined using FBA [9]. To predict the maximally possible bile acid production flux, the exchange reactions (in the single and pairwise models) and the fecal secretion reactions (in the community models) for CA, CDCA, and 13 secondary bile acids were individually chosen as the objective function and maximized. The 194 sample-specific community models were interrogated using distributedFBA [76]. To determine the total maximal production potential, the maximal flux through the fecal exchange reactions in the community models was maximized. To retrieve the contribution of each individual strain to overall production, the minimal fluxes through the luminal exchange reactions of each joined AGORA model (representing secretion into lumen) were extracted. Shadow prices were retrieved from each computed flux balance solution [7]. To extract the shadow prices for all metabolites in the respective community model that were computed while maximizing the production flux of secondary bile acids, a dedicated function (analyseObjectiveShadowPrices.m) in the Microbiome Modeling Toolbox [33] was used.

### Simulations

Model creation and contextualization, and simulations were carried out using the COBRA Toolbox [34] and the Microbiome Modeling Toolbox [33] in MATLAB version 2016b (Mathworks, Inc.) as programming environment. Flux balance analysis (FBA) [9] for pairwise simulations was performed using the optimization solver CPLEX through the Tomlab (Tomlab, Inc.) interface for MATLAB. Distributed flux balance analysis (distributedFBA) [76] for personalized microbiome simulations was performed using the IBM CPLEX solver (IBM, Inc.) through the CPLEX interface for Julia.

### Data analysis

The calculation of the Spearman correlation and the two-sided Wilcoxon rank-sum test was performed in MATLAB version 2016b (Mathworks, Inc.) using the corr and ranksum functions, respectively. The *p* values were adjusted for false-positive discovery rate with the Benjamini-Hochberg method using the mafdr function in MATLAB. Heatmaps were generated with R version 3.3.2 [77] using the aheatmap, pheatmap, ggplot2, easyGgplot2, and RColorBrewer packages, as well as the ComplexHeatmap package in Bioconductor 2.7 (http://www.bioconductor.org/). Principal Coordinates Analysis (PCoA) was performed with the vegan package in R using the Bray-Curtis dissimilarity index. Other plots were generated with MATLAB.

## Additional files

Additional file 1: **Table S1.** Bile acid deconjugation and transformation genes identified in the 693 analyzed genomes. **Table S2.** Description of the bile acid metabolism subsystem reconstructed for AGORA: a) metabolites, b) reactions. **Table S3.** Constraints implemented to simulate the Average European (AE) diet supplemented with taurocholate, glycocholate, taurochenodeoxycholate, and glycochenodeoxycholate. **Table S4.** Bile acid production potential for each of the 232 AGORA models carrying bile acid reactions. **Table S5.** Secondary bile acids produced in pairwise AGORA models that could not be produced individually by either model. **Table S6.** Bile acid production potential (mmol × person^-1^ × day^-1^) predicted for the community models representing 194 human microbiomes. **Table S7.** Features that significantly differed between 15 pediatric Crohn's Disease patients and 25 healthy controls. **Table S9.** Correlations between total community bile acid production (mmol × person^-1^ × day^-1^) and total community reaction abundance across all 194 community models. **Table S10.** Shadow prices in the flux balance solutions when optimizing for secondary bile acid production in all community models. Shown are metabolites that had a nonzero shadow price in at least one model. **Table S11.** Description of newly reconstructed strains in this study as an update to the AGORA resource. (XLSX 2194 kb)

Additional file 2: **Figure S1.** Maximum-likelihood phylogenetic tree for bile salt hydrolase (BSH) proteins and their homologs in 693 analyzed human gut microbe genomes. **Figure S2.** Maximum-likelihood phylogenetic tree for hydroxysteroid dehydrogenase (HSDH)/ bai cluster proteins and their homologs in 693 analyzed human gut microbe genomes. **Figure S3.** Genomic organization of baiNOP containing loci. **Figure S4.** Maximal-likelihood phylogenetic tree for homologs of the BaiN protein in 693 analyzed human gut microbe genomes. **Figure S5.** Maximal-likelihood phylogenetic tree for homologs of the BaiO protein in 693 analyzed human gut microbe genomes. **Figure S6.** Maximal-likelihood phylogenetic tree for homologs of the BaiP protein in 693 analyzed human gut microbe genomes. **Figure S7.** Heat map of the strain-level contributions clustered in Fig. 3d, and presented in Additional file 1: Table S8. (DOCX 3573 kb)
