# Supplementary material for: Systematic assessment of secondary bile acid metabolism in gut microbes reveals distinct metabolic capabilities in inflammatory bowel disease
Source: Microbiome. 2019 May 15;7:75. doi: 10.1186/s40168-019-0689-3 (PMC6521386; doi:10.1186/s40168-019-0689-3)
Supplement: Supplementary file 2 — Figure S1. Maximum-likelihood phylogenetic tree for bile salt hydrolase (BSH) proteins and their homologs in 693 analyzed human gut microbe genomes. Figure S2. Maximum-likelihood phylogenetic tree for hydroxysteroid dehydrogenase (HSDH)/ bai cluster proteins and their homologs in 693 analyzed human gut microbe genomes. Figure S3. Genomic organization of baiNOP containing loci. Figure S4. Maximal-likelihood phylogenetic tree for homologs of the BaiN protein in 693 analyzed human gut microbe genomes. Figure S5. Maximal-likelihood phylogenetic tree for homologs of the BaiO protein in 693 analyzed human gut microbe genomes. Figure S6. Maximal-likelihood phylogenetic tree for homologs of the BaiP protein in 693 analyzed human gut microbe genomes. Figure S7. Heat map of the strain-level contributions clustered in Fig. 3d, and presented in Additional file 1: Table S8. (DOCX 3573 kb) [file 40168_2019_689_MOESM2_ESM.docx]

**Supplementary Figures for**

**Systematic assessment of secondary bile acid metabolism in gut microbes reveals distinct metabolic capabilities in inflammatory bowel disease**

Almut Heinken^1^, Dmitry A. Ravcheev^1^, Federico Baldini^2^, Laurent Heirendt^2^, Ronan M.T. Fleming^3^, and Ines Thiele^1, 2,4*^

^1^School of Medicine, National University of Ireland, Galway, University Road, Galway, Ireland

^2^Luxembourg Centre for Systems Biomedicine, University of Luxembourg, Belvaux, Luxembourg

^3^Division of Analytical Biosciences, Leiden Academic Centre for Drug Research, Faculty of Science, University of Leiden, Leiden, The Netherlands

^4^Discipline of Microbiology, School of Natural Sciences, National University of Ireland, Galway, University Road, Galway, Ireland

*Corresponding author: Ines Thiele, School of Medicine, National University of Ireland, Galway, University Road, Galway, Ireland


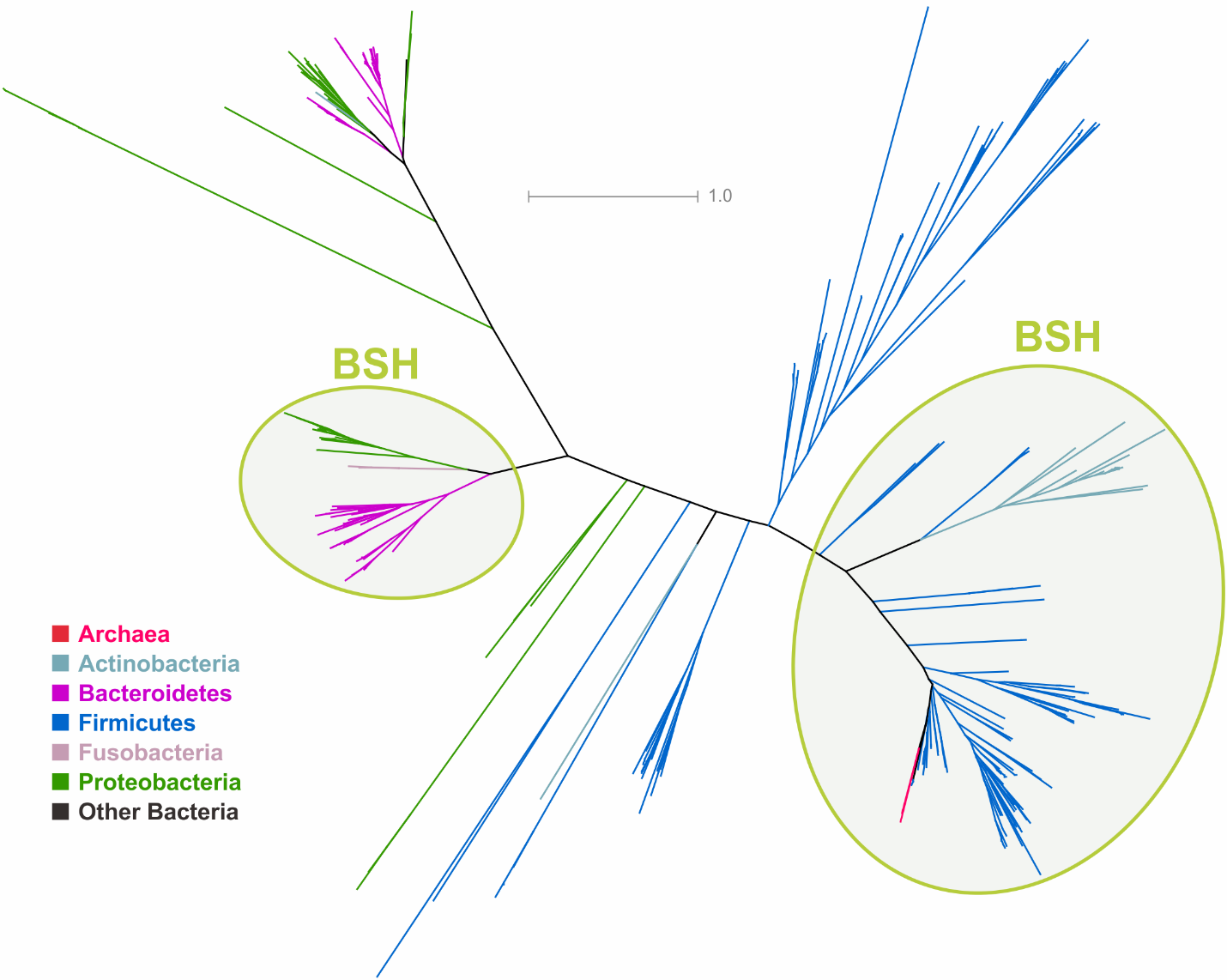


**Figure S1**: Maximum-likelihood phylogenetic tree for bile salt hydrolase (BSH) proteins and their homologs in 693 analyzed human gut microbe genomes. Branches are colored in agreement with microbial phyla. Two branches corresponding to the BSH proteins are shown in green ellipses.


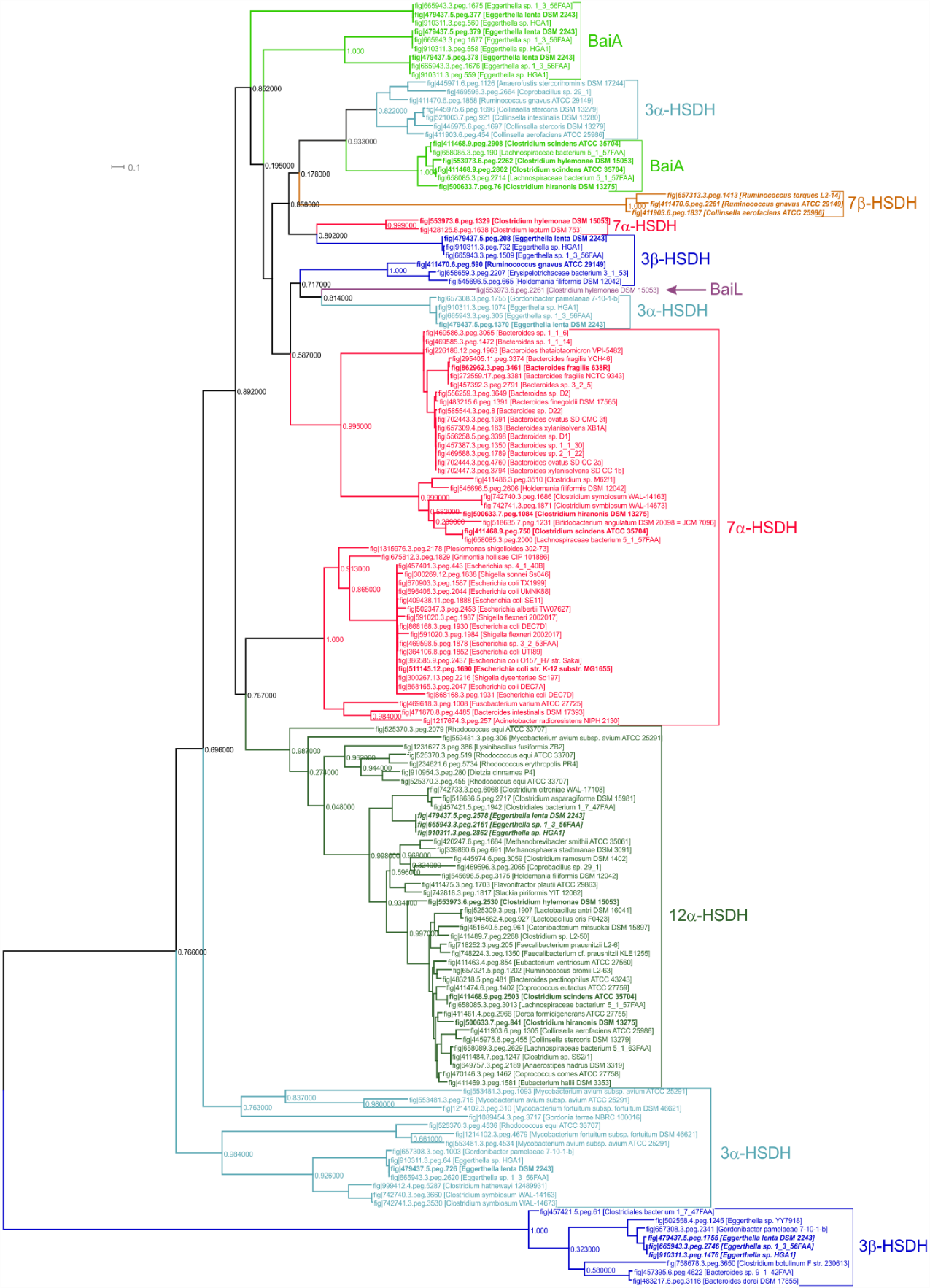


**Figure S2**: Maximum-likelihood phylogenetic tree for hydroxysteroid dehydrogenase (HSDH)/ *bai* cluster proteins and their homologs in 693 analyzed human gut microbe genomes. Colors of branches correspond to functions of the proteins.


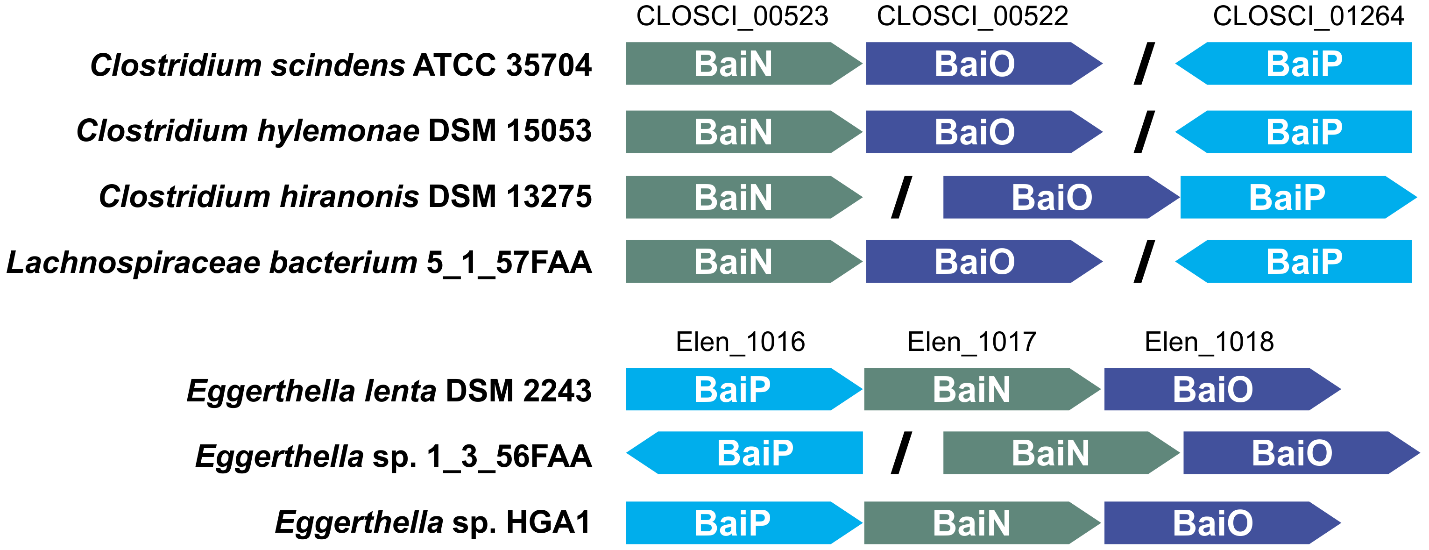


**Figure S3**: Genomic organization of *baiNOP* containing loci. Different loci are separated by slashes. Locus tags are shown for *C. scindens* ATCC 35704 and *E. lenta* DSM 2243.


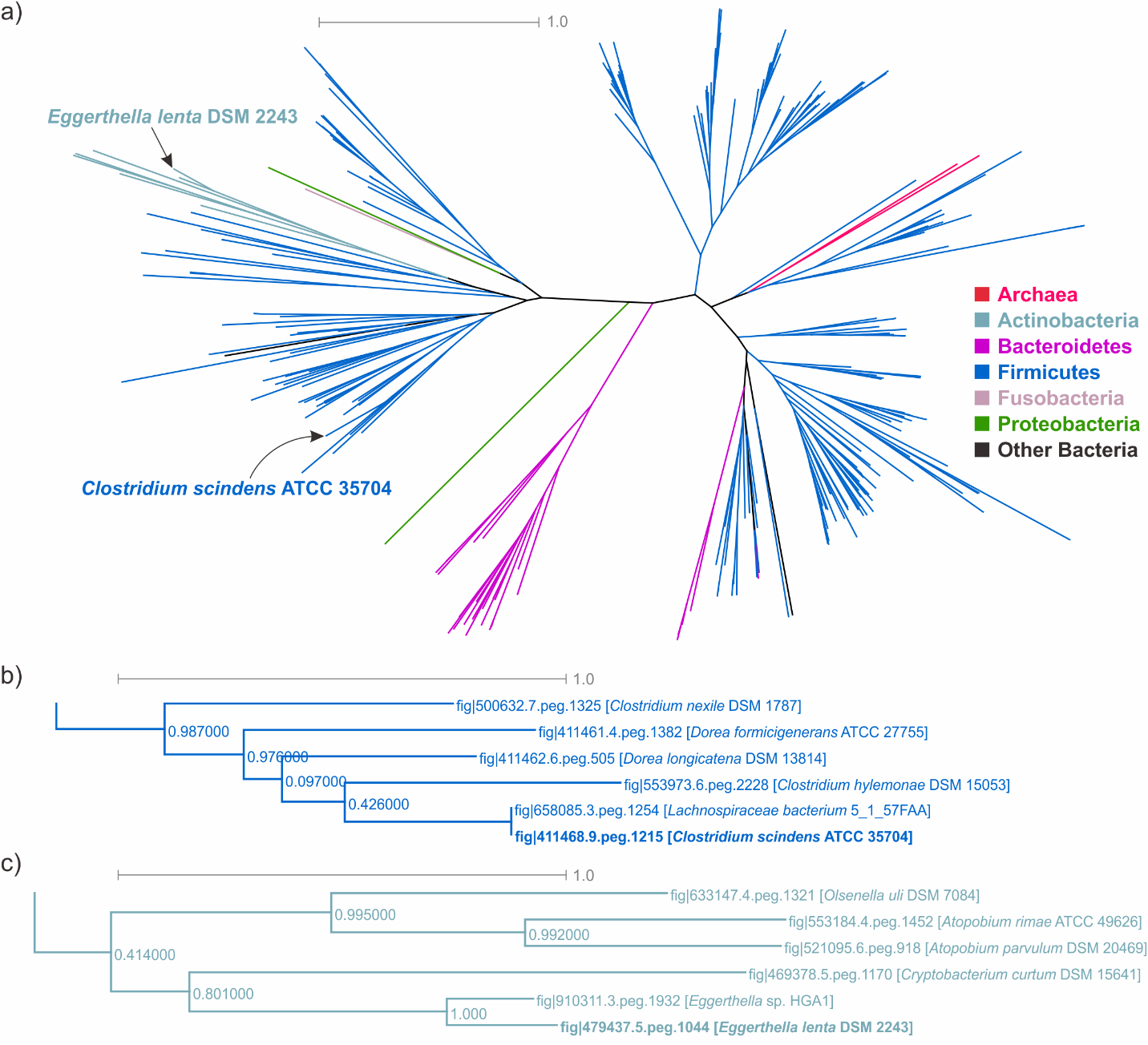


**Figure S4**: Maximal-likelihood phylogenetic tree for homologs of the BaiN protein in 693 analyzed human gut microbe genomes. Wide scale phylogeny for 317 proteins, branches are colored in agreement with microbial phyla (a). Details of the region of the tree containing proteins from *C. scindens* (b) and *E. lenta* (c), corresponding proteins are shown in bold.


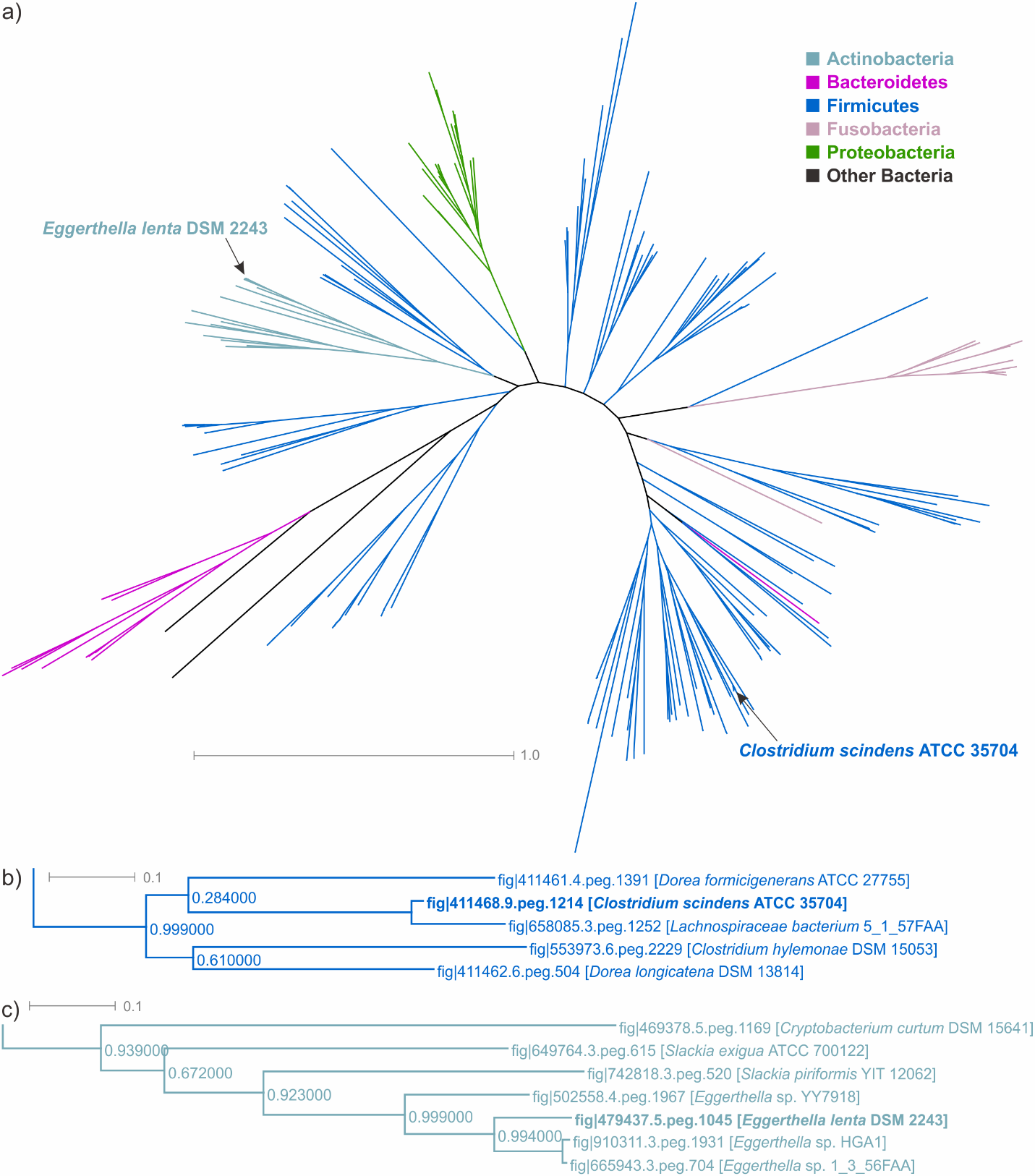


**Figure S5**: Maximal-likelihood phylogenetic tree for homologs of the BaiO protein in 693 analyzed human gut microbe genomes. Wide scale phylogeny for 201 proteins, branches are colored in agreement with microbial phyla (a). Details of the region of the tree containing proteins from *C. scindens* (b) and *E. lenta* (c), corresponding proteins are shown in bold.


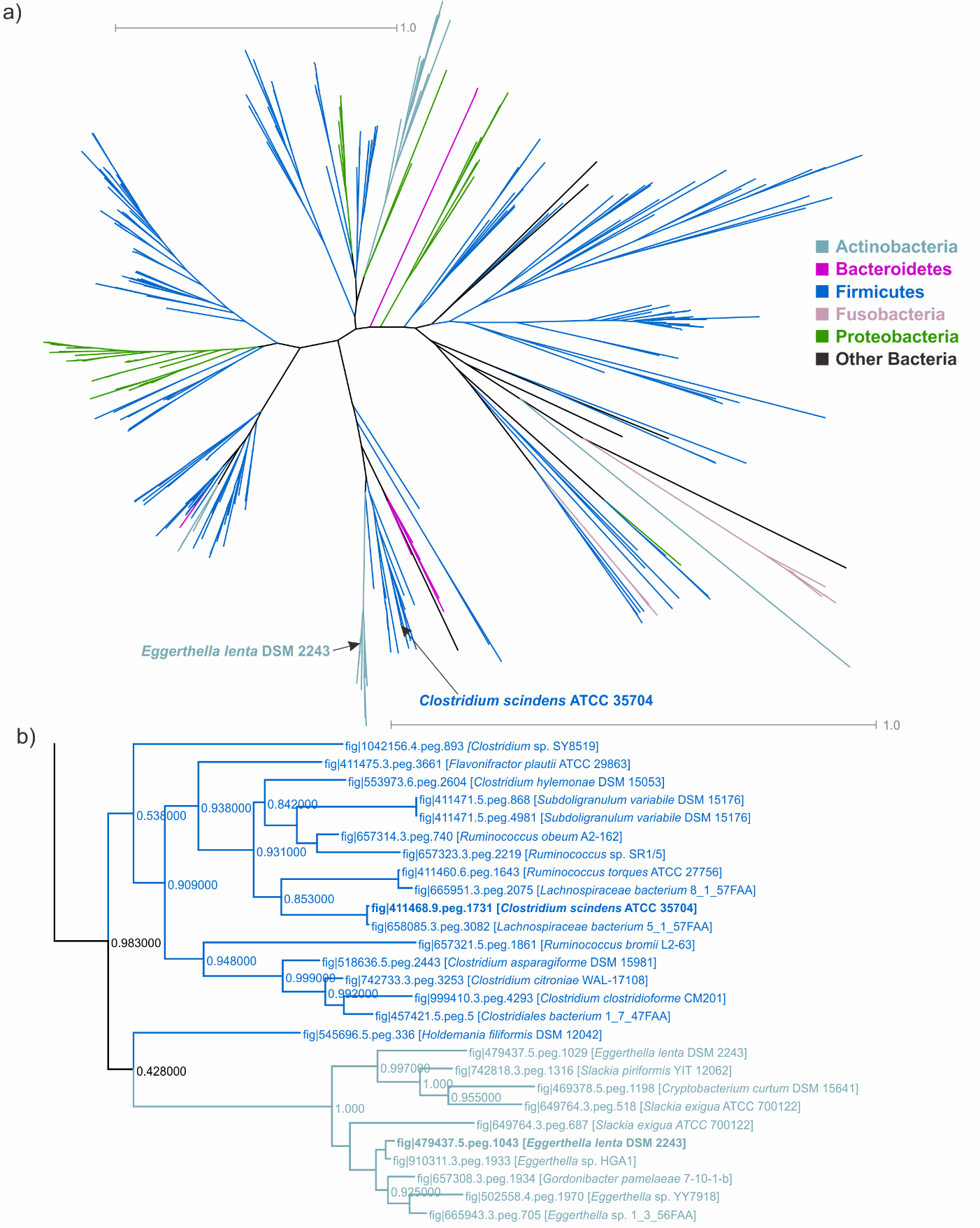


**Figure S6**: Maximal-likelihood phylogenetic tree for homologs of the BaiP protein in 693 analyzed human gut microbe genomes. Wide scale phylogeny for 335 proteins, branches are colored in agreement with microbial phyla (a). Details of the region of the tree containing proteins from *C. scindens* and *E. lenta* (b) corresponding proteins are shown in bold.


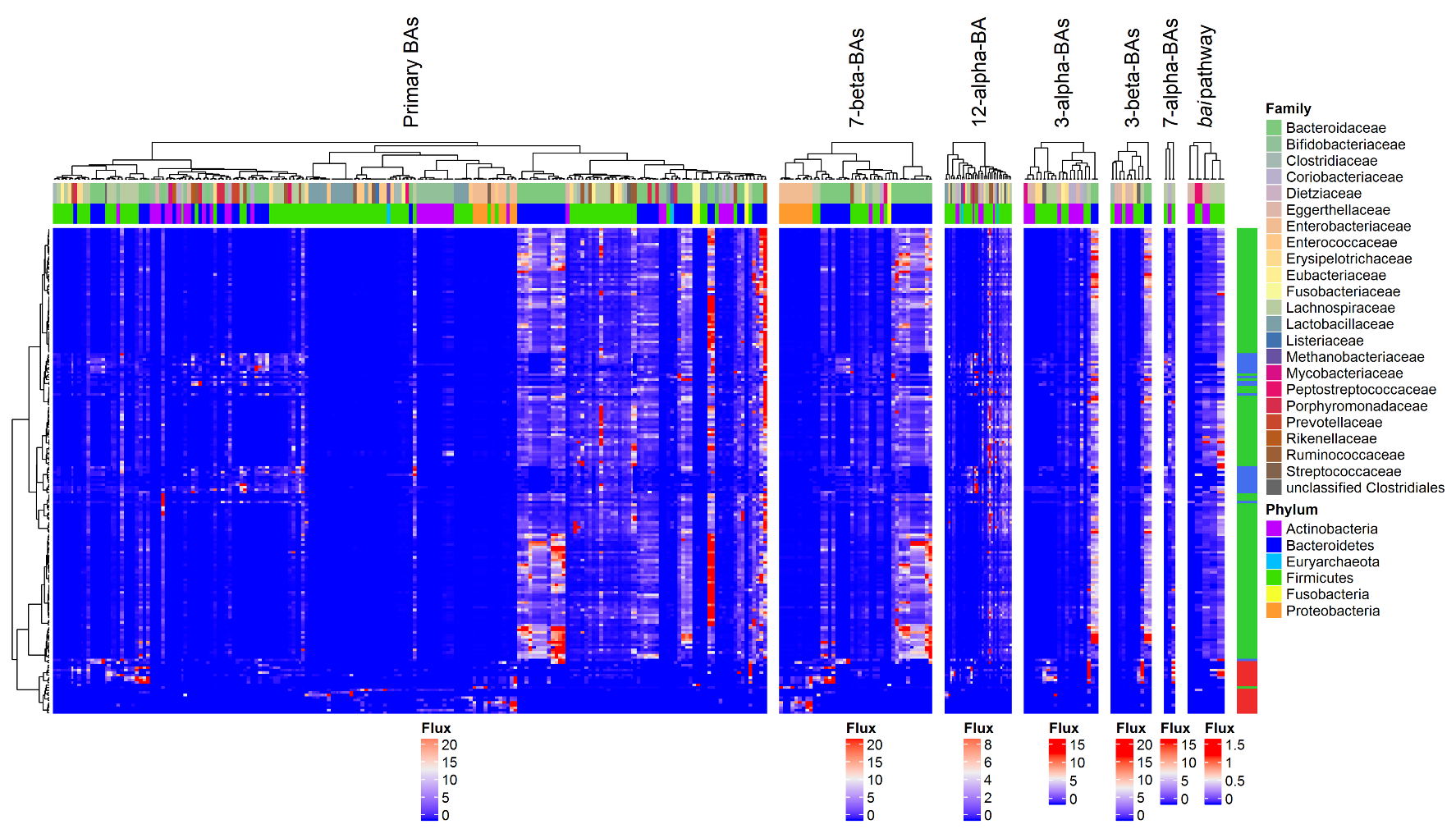


**Figure S7:** Heat map of the strain-level contributions clustered in Figure 3d) and presented in Additional file 1, Table S8. The columns show all strains that contributed in at least one microbiome annotated by family and phylum. The rows show the 194 microbiomes annotated by group. For metabolite abbreviations see Table 1.
